# The Inhibition of Fibrosis and Inflammation in Obstructive Kidney Injury via the miR-122-5p/SOX2 Axis Using USC-Exos

**DOI:** 10.34133/bmr.0013

**Published:** 2024-04-10

**Authors:** Wenjun Lu, Yujun Guo, Hengchen Liu, Tingting Zhang, Mingzhao Zhang, Xiangqi Li, Zhou Li, Manyu Shi, Zhitao Jiang, Zheng Zhao, Shulong Yang, Zhaozhu Li

**Affiliations:** ^1^ Department of Pediatric Surgery, The Sixth Hospital Affiliated to Harbin Medical University, Harbin Medical University, No.998 Aiying Street, Harbin 150027, Heilongjiang, China.; ^2^ Key Laboratory of Growth Regulation and Translational Research of Zhejiang Province, School of Life Sciences, Westlake University,Hangzhou 310024, Zhejiang, China.; ^3^ Center for Infectious Disease Research, Westlake Laboratory of Life Sciences and Biomedicine, Hangzhou 310024, Zhejiang, China.; ^4^ Laboratory of Systems Immunology, Institute of Basic Medical Sciences, Westlake Institute for Advanced Study, Hangzhou 310024, Zhejiang, China.; ^5^ Department of General Surgery, The Second Hospital Affiliated to Zhejiang University School of Medicine, No. 88 Jiefang Road, Hangzhou 310022, Zhejiang, China.; ^6^ Department of General Surgery, The Second Hospital Affiliated to Anhui Medical University, No. 678 Furong Road, Hefei 230031, Anhui, China.

## Abstract

**Background:** Fibrosis and inflammation due to ureteropelvic junction obstruction substantially contributes to poor renal function. Urine-derived stem-cell-derived exosomes (USC-Exos) have therapeutic effects through paracrine. **Methods:** In vitro, the effects of USC-Exos on the biological functions of HK-2 and human umbilical vein endothelial cells were tested. Cell inflammation and fibrosis were induced by transforming growth factor-β1 and interleukin-1β, and their anti-inflammatory and antifibrotic effects were observed after exogenous addition of USC-Exos. Through high-throughput sequencing of microRNA in USC-Exos, the pathways and key microRNAs were selected. Then, the antifibrotic and anti-inflammatory effects of exosomal miR-122-5p and target genes were verified. The role of the miR-122-5p/SOX2 axis in anti-inflammatory and antifibrotic effects was verified. In vivo, a rabbit model of partial unilateral ureteral obstruction (PUUO) was established. Magnetic resonance imaging recorded the volume of the renal pelvis after modeling, and renal tissue was pathologically analyzed. **Results:** We examined the role of USC-Exos and their miR-122-5p content in obstructive kidney injury. These Exos exhibit antifibrotic and anti-inflammatory activities. SOX2 is the hub gene in PUUO and negatively related to renal function. We confirmed the binding relationship between miR-122-5p and SOX2. The anti-inflammatory and antifibrotic effects of miR-122-5p were inhibited, indicating that miR-122-5p has anti-inflammatory and antifibrotic effects by inhibiting SOX2 expression. In vivo, the PUUO group showed typical obstructive kidney injury after modeling. After USC-Exo treatment, the shape of the renal pelvis shown a remarkable improvement, and inflammation and fibrosis decreased. **Conclusions:** We confirmed that miR-122-5p from USC-Exos targeting SOX2 is a new molecular target for postoperative recovery treatment of obstructive kidney injury.

## Introduction

Congenital obstructive nephropathy is caused by structural abnormalities of the urinary system [1] and is the main cause of chronic kidney disease (CKD) in children [2]. The most common lesion location is the ureteropelvic junction (UPJ). The progression of the lesion involves renal interstitial inflammation and fibrosis, both of which can damage kidney function. Moreover, because congenital obstructive kidney disease is mostly caused by incomplete unilateral ureteral obstruction (UUO), many patients do not have typical symptoms in the early stages, leading to some older children who have already experienced a certain degree of renal interstitial fibrosis and renal dysfunction when seeking medical treatment. Given the unique nature of the child population, this study focuses more on the growth and long-term function optimization of the kidneys. Even after surgical removal of the obstruction [3], new treatment methods are needed to reduce the progression of renal injury. Although relieving obstruction has remarkable therapeutic effects on both acute kidney injury (AKI) and CKD caused by obstructive kidney disease, long-term renal sequelae after relieving obstruction may eventually result in progression to end-stage kidney disease [4].

In recent years, an increasing number of studies have shown that exosomes derived from stem cells have therapeutic potential for fibrosis and inflammation caused by obstructive kidney disease, especially exosome-derived microRNAs (miRNAs) [5–7]. Exosomes are extracellular vesicles with a diameter of 40 to 160 nm, produced through the multivesicular endosome pathway [8]. These vesicles act as carriers to transport biological substances from seed cells to target cells [9]. The contents of exosomes mainly include lipids, proteins [tumor susceptibility gene 101 (TSG101), heat shock protein 70 (HSP70), CD9, CD63, and CD81], RNA (miRNA, mRNA, and long noncoding RNA), and DNA [10,11].

miRNAs are often regarded as key factors regulating intercellular communication, and on the basis of the inhibitory effect of miRNAs on target mRNA gene expression, exosomes are used to deliver specific miRNAs, targeting damage or disease-related target genes to achieve precise molecular-targeted therapy for damage repair and disease [12,13]. Bone marrow mesenchymal stem cells (BMSCs) overexpressing miR-let-7c selectively localized in the damaged kidney and up-regulated the expression of miR-let-7c. Compared with the control treatment, miR-let-7c-BMSC treatment alleviated renal injury and remarkably down-regulated type IV collagen, matrix metalloproteinase-9, and transforming growth factor-β1 (TGF-β1), and TGF-β1 receptor expression in kidneys with UUO [14]. Adipose-derived mesenchymal-stem-cell-derived exosomes improved diabetic nephropathy by transferring miR-26a-5p to high-glucose-induced mouse glomerular podocytes (MPC5), improving the viability of MPC5 cells, while inhibiting their apoptosis [15].

Urine-derived stem cells (USCs) are a newly discovered type of stem cell that was first reported by Zhang et al. [16] in 2008. They are a subset of cells with mesenchymal stem cell characteristics isolated from urine. Compared to other mesenchymal stem cells, USCs exhibit better potential to differentiate into urinary system tissue [17–19]. USCs can serve as an ideal source of seed cells for tissue damage repair, mainly due to their simple, low-cost, and noninvasive acquisition procedures [20]. With continuous in-depth research, miRNAs have been found to be key molecules in the therapeutic e ffect of USC-Exos. USC-Exos can protect against AKI through exosomal miR-146a-5p, which targets the 3′ untranslated region (UTR) of interleukin-1 (IL-1) receptor-associated kinase 1 and subsequently inhibits nuclear factor κB signaling and infiltration of inflammatory cells to protect renal function [21]. The application of USC-Exos in obstructive kidney injury has not yet been reported.

However, neither the UUO or ischemia–reperfusion injury models used in AKI studies nor the drug-induced (gentamicin or streptozotocin) models used in CKD studies are fully suitable for the pathological changes of UPJ obstruction (UPJO) [22,23]. Therefore, this study found that a partial UUO model (PUUO model) shows more similarities to the etiology and pathological changes of UPJO. This study aims to investigate whether USC-Exos and exosomal miR-122-5p have an active therapeutic effect on renal fibrosis and inflammation induced by PUUO/TGFβ1 + IL-1β in vivo/in vitro. Through screening data from the Nephroseq V5/GEO (Gene Expression Omnibus) database and predicting the binding sites of miR-122-5p target genes in the miRTarBase database, as well as experimental validation in this study, we found that SOX2 may be a negative regulatory factor related to obstructive kidney injury, and miR-122-5p can specifically bind to SOX2, exerting therapeutic effects by targeting SOX2 to inhibit its expression. miRNA delivery based on exosomes may provide a new molecular targeted therapeutic approach for the treatment of obstructive kidney injury.

## Methods

### Cell culture and identification of USCs

Fresh clean middle urine samples were obtained from 10 healthy volunteers. A total of 200 ml of urine from each donor was obtained in one experiment. The sample was centrifuged at 400*g* for 10 min and then washed once with phosphate-buffered saline (PBS). The cells were seeded in 6-well plates and allowed to grow for 7 d. By this time, the cell colonies could be observed, and USCs were passaged and expanded after another week. The cells were used for subsequent experiments when they were subcultured to passages 3 to 5 (P3 to P5). USCs were cultured in high-glucose Dulbecco’s modified Eagle medium and renal epithelial cell growth medium with 10% fetal bovine serum and 1% penicillin/streptomycin at 37 °C and 5% CO_2_. HK-2 cells and human umbilical vein endothelial cells (HUVECs) were purchased from the National Collection of Authenticated Cell Cultures (Shanghai, China). HK-2 cells were cultured in Dulbecco’s modified Eagle’s medium/F12 (Gibco, USA) with 10% fetal bovine serum and 1% penicillin/streptomycin at 37 °C and 5% CO_2_. HUVECs were cultured in RPMI 1640 medium with 10% fetal bovine serum and 1% penicillin/streptomycin at 37 °C and 5% CO_2_. The identification of surface markers, which involved fluorescein isothiocyanate (FITC)-conjugated antibodies against CD73, CD90, CD34, and CD45, phycoerythrin (PE)-conjugated antibody against CD146, and Alexa-Fluor-488-conjugated antibody against human leukocyte antigen (HLA)-DR (BioLegend, San Diego, USA), was performed by flow cytometry. Pluripotency markers and renal markers, including Nano G (monoclonal; 1:1,000; ab109250, Abcam, Cambridge, UK), anti-Wilm’s tumor-1 (anti-WT-1; monoclonal; 1:1,000; ab267377, Abcam), and nephrin (anti-nephrin; polyclonal; 1:1,000; ab235903, Abcam), were detected by Western blotting. The multilineage differentiation of USCs was determined using adipogenic, osteogenic, and chondrogenic medium and examined by Oil red O staining, Alizarin red staining, and toluidine blue staining.

### Isolation, characterization, and tracing of exosomes

The isolation of exosomes was performed as in a previous study [24]. Exosomes were resuspended in PBS after removing the supernatant. Nanoparticle tracking analysis, transmission electron microscopy (TEM), and Western blotting were used for the identification of exosomes. For confirmation that exosomes can be absorbed by target cells/tissues in vitro/in vivo. USC-Exos were incubated with 1 μM phycoerythrin-conjugated hexadecylamine 26/1,1'-dioctadecyl-3,3,3',3'-tetramethylindotricarbocyanine iodide (PKH26/DiR) (Sigma-Aldrich, St. Louis, MO, USA) in Diluent C (Sigma-Aldrich) for 5 min, and excess dye was removed. The PKH26/DiR fluorescently labeled USC-Exos were subsequently added to the serum-free medium of HK-2 cultures and incubated overnight, and the supernatants were used to isolate PKH26/DiR-labeled USC-Exos in the same procedure as above. The nuclei were labeled with Hoechst 33342 (UE, China). The DiR-labeled USC-Exos were intravenously administered to experimental animals. In vitro tracing images were taken with an inverted fluorescence microscope (Leica, Wetzlar, Germany). Tracing in vivo was performed by an animal imaging system (NightOWL LB 983, Berthold Technologies Bioanalytics, Germany).

### In vitro treatment with USC-Exos/exosomal miR-122-5p

The concentration gradient was set to 0, 25, 50, and 100 μg/ml. We found that the proliferative, migration, antifibrotic, and anti-inflammatory abilities were strongest at a concentration of 100 μg/ml. Therefore, this concentration was uniformly selected in subsequent pathway validation. In vitro experimental inflammation and fibrosis models were combined with TGF-β1 and IL-1β at a concentration of 5 ng/ml. When adding pathway inhibitors to verify the downstream pathway of USC-Exos, we randomly divided the cells into 5 groups as follows: (a) control: exosome-free medium only; (b) USC-Exos: exosome-free medium containing USC-Exos (100 μg/ml); (c) USC-Exos + LY294002 (MCE, USA): exosome-free medium containing USC-Exos (100 μg/ml) and 10 nM phosphatidylinositol 3-kinase (PI3K)-AKT inhibitor; (d) USC-Exos + PD98059: exosome-free medium containing USC-Exos (100 μg/ml) and 10 nM extracellular signal–regulated kinase 1/2 (ERK1/2) inhibitor; and (e) USC-Exos + SB203580: exosome-free medium containing USC-Exos (100 μg/ml) and 10 nM p38 inhibitor. Pathway inhibitor was added to HK-2 cells 30 min earlier than USC-Exos. After the key miRNA of this experiment was identified as miR-122-5p, the transfection groups were NC (empty vector group), miR-122-5p mimic, inhibitor NC, and miR-122-5p inhibitor. After determination of the target of miR-122-5p as SOX2, the transfection groups were as follows: (a) NC, transfected with SOX2 overexpression plasmid; and (b) NC, miR-122-5p mimic, and cotransfection with miR-122-5p mimic and SOX2 overexpression plasmids.

### Cell proliferation assay

The thymine nucleoside analog 5-ethynyl-2′-deoxyuridine (EdU) can penetrate into replicating DNA during cell proliferation. Cell proliferation can be accurately reflected by detecting the combination of EdU and fluorescent dyes. HK-2 cells and HUVECs were incubated with 50 μM EdU from an EdU assay kit (UE) for 2 h. HK-2 cells and HUVECs were fixed with 4% paraformaldehyde. Then, the cells were stained and labeled with Click T mixture and Hoechst 33342 in an EdU assay kit. Images were taken with an inverted fluorescence microscope.

### Scratch wound assay

HK-2 cells and HUVECs at 2 × 10^5^ cells per well were seeded in a 6-well plate. When the cells grew to be fused into a monolayer, a straight-line wound was made on the fused monolayer cells using a sterile 200-μl pipette tip. Serum-free medium with different concentrations of USC-Exos and/or pathway inhibitors was then added to each well. Photos were taken and recorded at 2 time points of 0 and 24 h using an inverted microscope with an Axiocam 305 color digital camera and ZEN 2011 software (Carl Zeiss, Oberkochen, Germany).

### Transwell assay

HK-2 cells and HUVECs at 1 × 10^5^ cells per well were seeded in the transwell upper chamber, and USC-Exos and/or pathway inhibitors were added to the lower chamber. After culture for 24 h, HK-2 cells and HUVECs were fixed with 4% paraformaldehyde and stained with crystal violet. Images were obtained under a light taken with an Axiocam 305 color digital camera and ZEN 2011 software.

### Tube formation assay

HUVECs (1 × 10^5^ cells per well) were seeded on a 6-well plate containing Matrigel matrix (Corning, USA). This matrix was stored on ice at all times. Then, HUVECs were treated with different concentrations of USC-Exos (as mentioned before). After incubation at 37 °C with 5% CO_2_ for 2 h, images were acquired with an inverted microscope with an Axiocam 305 color digital camera and ZEN 2011 software. The total tube length and branch points were calculated by ImageJ software.

### Western blot analysis

HK-2 cells were lysed in radioimmunoprecipitation assay buffer (Beyotime, China). Immunoblotting was performed using the following rabbit primary antibodies: anti-CD9 (1:2,000; ab92726, Abcam, UK), anti-TSG101 (1:2,000; ab125011, Abcam), anti-HSP70 (1:1,000; ab2787, Abcam), anti-vimentin (1:1,000; ab92547, Abcam), anti-α-smooth muscle actin (anti-α-SMA; 1:1,000; ab7817, Abcam), anti-E-cadherin (anti-ECAD; 1:10,000; ab40772, Abcam), anti-cyclooxygenase 2 (anti-COX-2; 1:1,000; ab179800, Abcam), anti-AKT [1:1,000; 4691S, Cell Signaling Technology (CST), USA], anti-p-AKT (1:1,000; 4060S, CST), anti-mitogen-activated protein kinase (MAPK) (ERK1/2) (1:1,000; 8544S, CST), anti-p-MAPK (ERK1/2) (1:1,000; 8544S, CST), anti-p38 (1:1,000; 8690S, CST), anti-p-p38 (1:1,000; 9216S, CST), anti-SOX2 (1:1,000; AF5140, Affinity, Canada), and anti-β-actin (1:5,000; ab8226, Abcam). Horseradish-peroxidase-conjugated goat anti-rabbit immunoglobulin G (IgG) (1:5,000; BA1055, Boster, China) and horseradish-peroxidase-conjugated goat anti-rat IgG (1:5,000; S0009, Affinity, Canada) were used as the secondary antibodies. A chemiluminescence imaging system (ChemiScope 6200T, Clinx Science Instruments, Shanghai, China) was used for detection.

### miRNA isolation and high-throughput sequencing

miRNA high-throughput sequencing was performed by GENESKY Company (Shanghai, China). Exosomal total RNA was extracted using the miRNeasy Mini Kit (QIAGEN) for miRNA sequencing analysis. The quality and purity of the extracted RNA were determined using an Agilent 2100 Bioanalyzer (Agilent Technologies, USA). Total input of a single sample of ≥20 ng of total RNA, raw data of ≥10 M reads per sample, and a base ratio of Q30 > 80% meeting the above standards were sequenced and analyzed. The miRNA sequencing library was constructed using the TruSeq Small RNA sample Preparation Kit (Illumina, USA). We analyzed the expression of miRNAs using Illumina HiSeq 2500 (Illumina, USA) in the final step.

### Functional enrichment analysis

Gene Ontology (GO) and Kyoto Encyclopedia of Genes and Genomes (KEGG) pathway enrichment analyses of miRNAs identified through high-throughput sequencing were performed using the Database for Annotation, Visualization, and Integrated Discovery. The enrichment analysis was visualized by using R software.

### PUUO animal model

The PUUO model has been proven to be a reasonable model to study hydronephrosis caused by UPJO [25]. It was established using Japanese long-eared white rabbits (male; weight, 3,000 to 3,500 g) purchased from the animal experiment center of Harbin Medical University. After lying prone, the rabbits were anesthetized with intraperitoneal pentobarbital (4 mg/kg). A straight incision was made along the left side of the spine, the muscle space was passively separated, the ureter was found along the psoas major muscle, and a sterilized polyethylene plastic tube with a length of approximately 1 cm and an inner diameter of approximately 0.8 mm was cut longitudinally. Then, the ureter was inserted approximately 1 cm below the UPJ, ligated, and fixed at both ends with surgical sutures. Suturing after reduction of the ureter was performed. Antibiotics were injected intramuscularly for 3 d after surgery. After 2 weeks, we determined the formation of hydronephrosis by magnetic resonance imaging (MRI), and the cannula was removed. Then, the animals were randomly divided into 4 groups: (a) sham group: Only the abdomen was opened and closed without modeling (*n* = 6). (b) PUUO group: Examination should be conducted directly after 2 weeks of modeling (*n* = 6). (c) PBS treatment group: PBS with the same volume as USC-Exos was injected through the ear margin vein after removal of the cannula (*n* = 6). (d) USC-Exo treatment group: USC-Exos (400 μg/kg·d) were injected through the vein of ear margin after removal of the cannula (*n* = 6). In the latter 2 groups, the obstruction was relieved 2 weeks after modeling, and the animals were then treated for 1 week. MRI was performed, and euthanasia drugs were injected intravenously. Renal histopathology was assessed. Renal damage (glomerular atrophy and tubular dilatation) was scored according to the following criteria: 0 = damage area < 25%, 1 = damage area of 25% to 50%, 2 = damage area of 50% to 75%, and 3 = damage area > 75% [23].

### MRI examination

All rabbits were scanned by MRI at 2 weeks following the treatment to assess the presence of hydronephrosis and then scanned at 1 week after relieving obstruction. On the basis of the MRI images (sagittal, coronal, and transverse sections), the left renal pelvis volume (RPV) of each rabbit was quantified in accordance with a previously described method, following the calculation formula “maximum anteroposterior diameter” × “maximum length diameter” × “maximum transverse diameter” × 0.523 (Table 1) [26]. An Achieva 3.0T TX (Philips) MRI machine was used.

**Table 1.** Comparison of changes of renal pelvic volume among groups after injection (*X* ± *S*)

**Group**	**MAD (cm)**	**MLD (cm)**	**MTD (cm)**	**Renal pelvic volume (cm** ^**3**^ **)**
Sham	0.74 ± 0.13	1.68 ± 0.15	1.02 ± 0.12	0.67 ± 0.17
PUUO	2.01 ± 0.22	3.36 ± 0.22	2.79 ± 0.17	9.87 ± 1.28
PBS	1.85 ± 0.05	2.48 ± 0.37	2 ± 0.25	4.86 ± 1.36
USC-Exos	1.11 ± 0.09	2.15 ± 0.17	1.47 ± 0.11	1.82 ± 0.06

MAD, maximum anteroposterior diameter; MLD, maximum length diameter; MTD, maximum transverse diameter; renal pelvic volume, MAD × MLD × MTD × 0.523.

### Pathological assay

Paraffin-embedded renal tissues were sectioned at a thickness of 4 μm. The tissues were then stained with hematoxylin and eosin (H&E) and Masson for histopathological analysis. For immunohistochemical analysis, the renal tissues were incubated with Immuno-Block reagent for 30 min after being deparaffinized and rehydrated. The sections were then incubated with the following human primary antibodies: anti-TGF-β1 (1:500; ab215715, Abcam), anti-IL-1β (1:50; ab216995, Abcam), anti-COX-2 (1:100; ab179800, Abcam), anti-α-SMA (1:100; ab7817, Abcam), and anti-ECAD (1:500; ab40772, Abcam). Horseradish-peroxidase-conjugated goat anti-rabbit and goat anti-rat IgG (1:500; 115-035-003, Jackson ImmunoResearch, UK) was used as the secondary antibody. After counterstaining with hematoxylin, the sections were dehydrated and fixed. The area of the positive signal was determined using ImageJ software. For immunofluorescence analysis, the sections of renal tissues were incubated with the following human primary antibodies: anti-CD31 (1:100; ab9498, Abcam), anti-IL-6 (1:100; TA500067S, Origene), and anti-IL-10 (1:100; ab33471, Abcam). The sections were then incubated with secondary antibodies (1:200; SA00013, Proteintech, USA) for 1 h. The nuclei were labeled with 4′,6-diamidino-2-phenylindole, and images were taken with a DM4 B microscope (Leica). Three or 6 fields per section were selected randomly for statistical analysis. Positive signals were quantified with ImageJ software.

### Data processing of differentially expressed genes

The gene expression datasets analyzed in this study were from the GEO database. Only 2 datasets in the GEO database were carefully searched. The gene expression profiles are as follows: GSE45304 was based on the Agilent GPL7202 platform (Agilent-014868 Whole Mouse Genome Microarray 4x44K G4122F), and GSE96102 was based on the Agilent GPL4134 platform (Agilent-014868 Whole Mouse Genome Microarray 4x44K G4122F). All of the data are freely available online, and the miRNA expression profile in this study has already been uploaded to the Sequence Read Archive database (project no. PRJNA871972). The GEO2R online analysis tool was used to detect the differentially expressed genes (DEGs) between PUUO and sham/normal samples, and the adjusted *P* value and |logFC| were calculated. Genes that met the cutoff criteria, adjusted *P* < 0.05 and |logFC| ≥ 0.3, were considered DEGs. Statistical analysis was carried out for each dataset, and the intersecting part was identified using the Venn diagram webtool.

### Clinicopathological correlation analysis

Clinical characteristic data related to SOX2 and kidney disease were obtained from the Nephroseq V5 database. Pearson’s correlation analysis between SOX2 and glomerular filtration rate (GFR), urinary protein, and serum creatinine in patients with CKD was performed using the Nephroseq v5 online database. The statistical analysis was carried out using GraphPad Prism 9.0 (GraphPad Software Inc., La Jolla, CA, USA).

### Protein–protein interaction network

The protein–protein interaction (PPI) data of miRNA with the top 20 target genes and intersecting genes between GSE45304 and GSE96102 were analyzed by STRING (The Search Tool for the Retrieval of Interacting Genes). The screening of miRNA target genes was based on the miRDB, miRTarBase, and TargetScan databases by running Perl script. The PPI network and relationship between miRNAs and target genes were drawn by Cytoscape software and sorted by degree value. Nodes with a higher degree of connectivity tend to be more essential in maintaining the stability of the entire network. CytoHubba, a plugin in Cytoscape, was used to calculate the degree of each protein node. In this study, the top 50 miRNA target genes and top 10 genes of GSE45304 and GSE96102 were identified as hub genes.

### Dual luciferase reporter assay

Human embryonic kidney 293T cells (1 × 10^5^ cells per well) were seeded on a 24-well plate, and transfection was performed when the cells achieved 70% to 80% confluency. Thirty minutes before transfection, the complete medium was replaced with serum-free medium. Lipofectamine 3000 (CN2507605, Invitrogen by Thermo Fisher Scientific) was used to transfect the cells. The cells were lysed 48 h after transfection, and miRNA expression was detected by chemiluminescence using the Dual-Luciferase Reporter Assay System (Beyotime). With *Renilla* luciferase as the internal reference, the fluorescence ratio was calculated by dividing the relative light units for firefly luciferase by the relative light units for Renilla luciferase. The inhibitory effect of the miRNA on target gene expression was compared according to the obtained ratio.

### Quantitative real-time polymerase chain reaction

Total RNA was extracted from cells/tissues using a fast total RNA extraction kit (Seven Biotech, China). Quantitative real-time polymerase chain reaction (qRT-PCR) was performed using 2× SYBR Green qPCR Master Mix (Seven Biotech) according to the manufacturer’s instructions. The reaction volumes contained 1 μl of diluted cDNA solution, 5 μl of SYBR Green, and 0.5 μl each of forward, reverse and RT primer, and double-distilled H_2_O was added to replenish the volume to 10 μl. qRT-PCR was performed on a CFX96 touch (Bio-Rad) with the following cycling scheme: 5 min at 95 °C followed by 40 cycles of 15 s at 95 °C, 25 s at 60 °C, 1 s at 60 °C, and 1 s at 95 °C. *C*_t_ values were calculated with automatically set thresholds and baselines, and those higher than 30 were excluded from the analysis. The primers used for qRT-PCR are listed in Table 2.

**Table 2.** Specific primers used for qRT-PCR analysis

Gene symbol	Specific primers
hsa-miR-122-5p	5′ CGCGTGGAGTGTGACAATGG 3′ (forward) 5′ AGTGCAGGGTCCGAGGTATT 3′ (reverse) 5′ GTCGTATCCAGTGCAGGGTCCGAGGTATTCGCACTGGATACGACCAAACA 3′ (RT)
hsa-let-7a-5p	5′ GCGCGTGAGGTAGTAGGTTGT 3′ (forward) 5′ AGTGCAGGGTCCGAGGTATT 3′ (reverse) 5′ GTCGTATCCAGTGCAGGGTCCGAGGTATTCGCACTGGATACGAACAA 3′ (RT)
hsa-miR-30a-5p	5′ CGCGTGTAAACATCCTCGAC 3′ (forward) 5′ AGTGCAGGGTCCGAGGTATT 3′ (reverse) 5′ GTCGTATCCAGTGCAGGGTCCGAGGTATTCGCACTGGATACGACCT 3' (RT)
hsa-miR-10a-5p	5′ CGCGTACCCTGTAGATCCGAA 3′ (forward) 5′ AGTGCAGGGTCCGAGGTATT 3′ (reverse) 5′ GTCGTATCCAGTGCAGGGTCCGAGGTATTCGCACTGGATACGACCA 3′ (RT)
hsa-miR-10b-5p	5′ CGCGTACCCTGTAGAACCGAA 3′ (forward) 5′ AGTGCAGGGTCCGAGGTATT 3′ (reverse) 5′ GTCGTATCCAGTGCAGGGTCCGAGGTATTCGCACTGGATACGACCACAAA 3′ (RT)
hsa-miR-26a-5p	5′ CGCGTTCAAGTAATCCAGGA 3′ (forward) 5′ AGTGCAGGGTCCGAGGTATT 3′ (reverse)5′ GTCGTATCCAGTGCAGGGTCCGAGGTATTCGCACTGGATACGACAG 3′ (RT)
hsa-miR-200b-3p	5′ GCGCGTAATACTGCCTGGTAA 3′ (forward) 5′ AGTGCAGGGTCCGAGGTATT 3′ (reverse) 5′ GTCGTATCCAGTGCAGGGTCCGAGGTATTCGCACTGGATACGACTC 3′ (RT)
U6	5′ CTCGCTTCGGCAGCACATATACT 3′ (forward) 5′ ACGCTTCACGAATTTGCGTGTC 3′ (reverse) 5′ AAAATATGGAACGCTTCACGAATTTG 3′ (RT)
SOX2	5′ ATGGACAGTTACGCGCACAT 3′ (forward) 5′ CGAGCTGGTCATGGAGTTGT 3′ (reverse)
α-SMA	5′ CACCATCACCCCCTGATGTC 3′ (forward) 5′ ACTGCCTTGGTGTGTGACAA 3′ (reverse)
COX-2	5′ TGCGCCTTTTCAAGGATGGA 3′ (forward) 5′ ACCGTAGATGCTCAGGGACT 3′ (reverse)
GAPDH	5′ CTGGGCTACACTGAGCACC 3′ (forward) 5′ AAGTGGTCGTTGAGGGCAATG 3′ (reverse)

### Statistical analysis

All data are expressed as the means ± SD. The mean value of each group was analyzed and compared by ordinary one-way analysis of variance (ANOVA) and Tukey’s multiple comparisons test. *P < 0.05, **P < 0.01, ***P < 0.001, and ****P < 0.0001 indicate statistical significance.

## Results

### Characterization of USCs

**Fig. 1.**
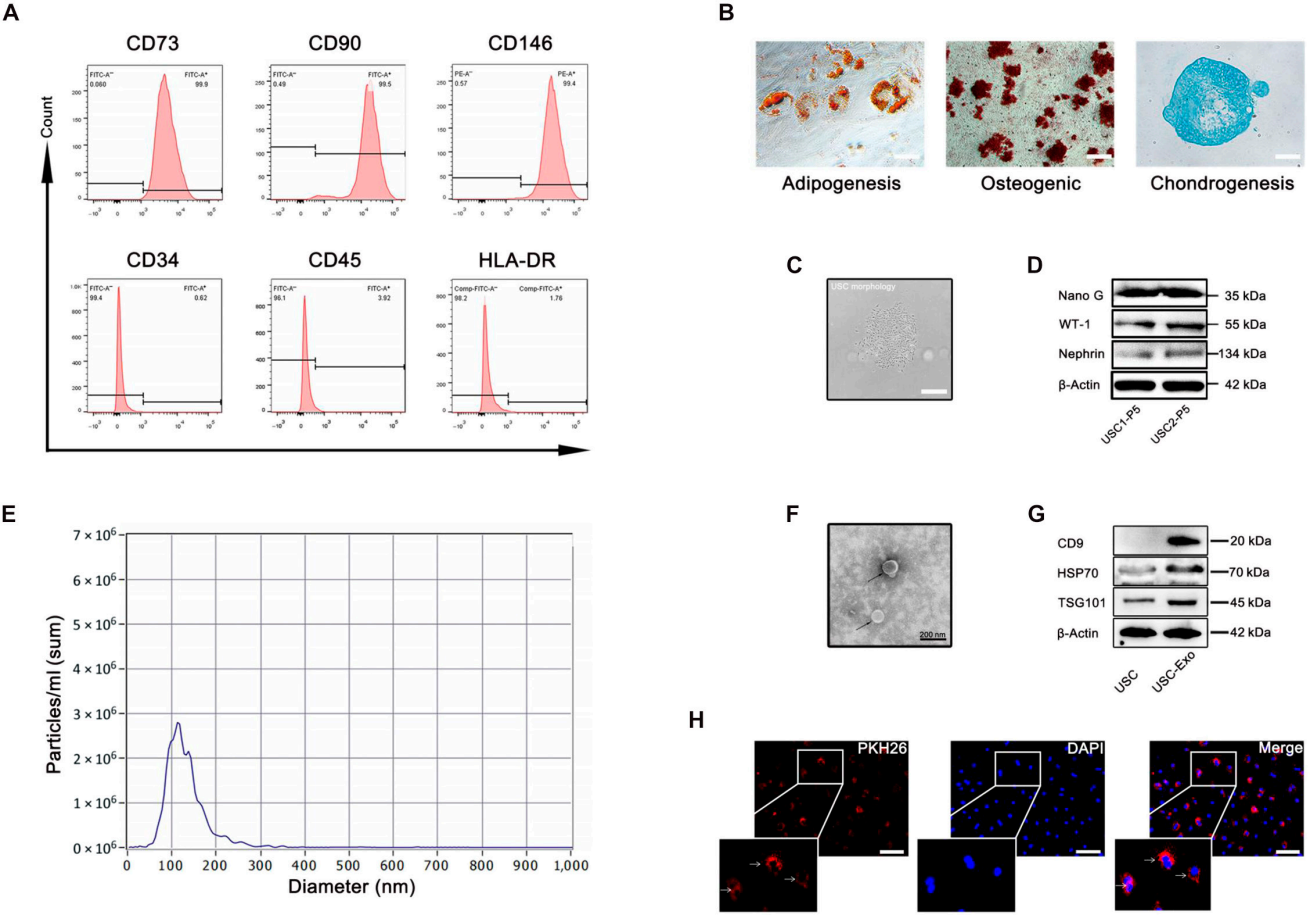
USC and USC-Exo phenotype and tracing. (A) Flow cytometry for positive/negative USC surface markers. (B) Adipogenic/osteogenic/chondrogenic differentiation potential of USCs. Scale bars, 100 μm. (C) Morphology of primary USCs. Scale bars, 100 μm. (D) Western blotting analysis of the pluripotency-related protein Nano G, the renal-derived related protein WT-1, and nephrin. (E) Nanoparticle tracking analysis. (F) Morphology of USC-Exos under TEM. Scale bars, 200 nm. (G) Exosome surface markers detected by Western blotting. (H) PKH26-labeled USC-Exo internalization by HK-2. Scale bars, 100 μm. DAPI, 4′,6-diamidino-2-phenylindole.

Flow cytometry was used to detect common surface markers of mesenchymal stem cells, which were CD73-, CD146-, and CD90-positive and CD34- and CD45-negative cells (Fig. 1A). HLA-DR, a marker of low immunogenicity, was also found (Fig. 1A). USCs could differentiate into adipocytes, osteoblasts, and chondroblasts in vitro (Fig. 1B). Primary USCs were cultured for 5 to 7 d, and a few tiny colonies could be observed, with spindle-shaped cells (Fig. 1C). Pluripotency markers of USCs included Nano G (Fig. 1D). Nano G is an endogenous transcription factor that plays an important role in maintaining stem cell pluripotency [27,28]. Western blot analysis proved that USCs expressed special renal markers, including WT-1 and nephrin (Fig. 1D), suggesting a possible renal source of USCs.

### Characterization and tracing of USC-Exos

Nanoparticle tracking analysis revealed the mean diameter of USC-Exos to be 112.7 nm (Fig. 1E). TEM revealed that USC-Exos were round or elliptical vesicular structures (Fig. 1F). Western blot analysis confirmed that the USC-Exo surface markers CD9, TSG101, and HSP70 (Fig. 1G). In addition, PKH26-labeled USC-Exos surrounded the nucleus of HK-2 cells and showed red fluorescence (Fig. 1H).

### USC-Exos promote proliferation and migration of HK-2 cells

**Fig. 2.**
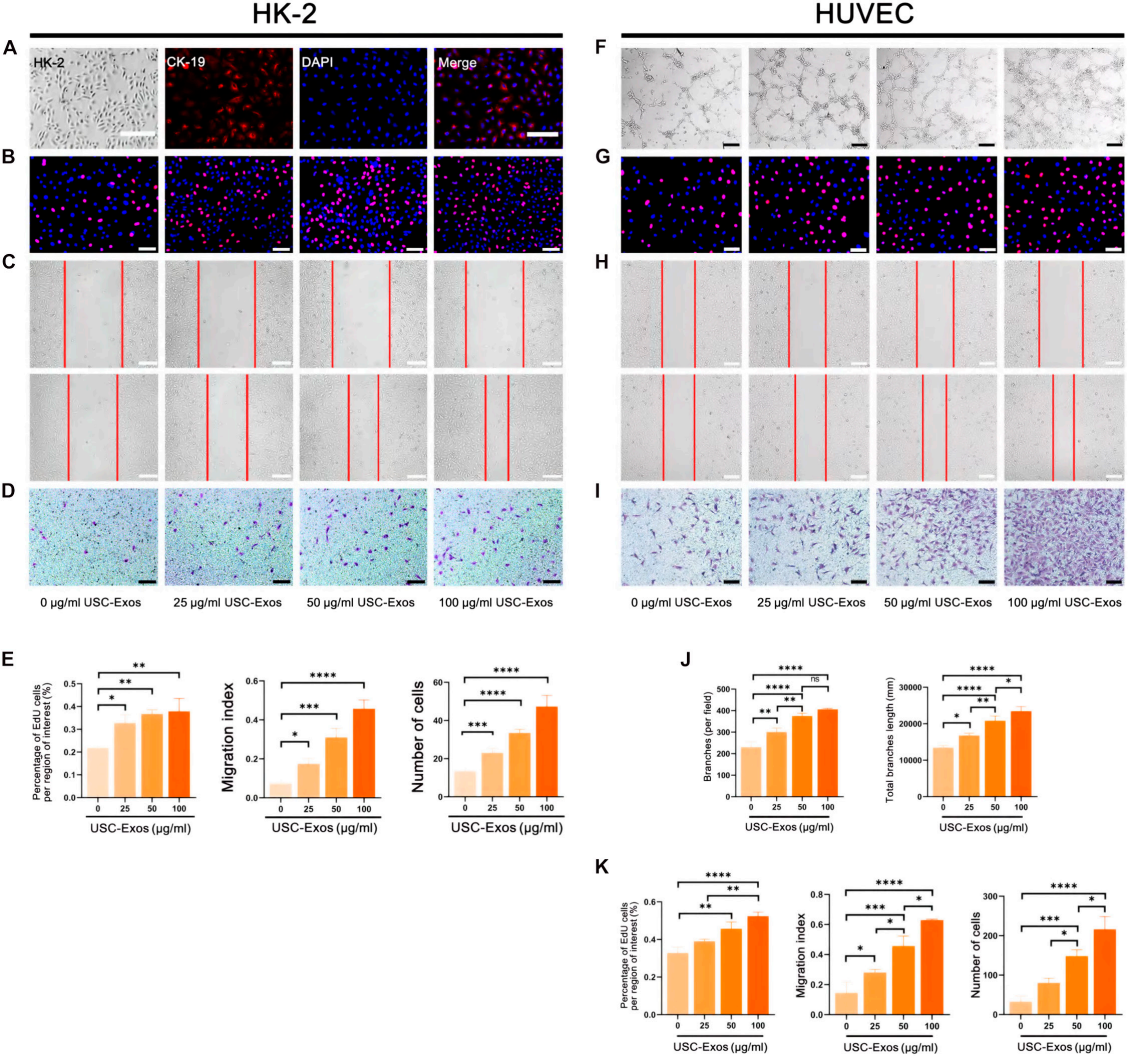
USC-Exos promoted proliferation, migration, and angiogenesis with increasing concentrations. (A) CK-19-labeled HK-2. (B and G) Effect of different concentrations of USC-Exos on the proliferation of HK-2 cells and HUVECs by EdU assays. (C, D, H, and I) Effect of different concentrations of USC-Exos on the migration of HK-2 cells and HUVECs by transwell assays and scratch assays. (E and K) Columnar statistics for proliferation and migration. (F) Effect of different concentrations of USC-Exos on the angiogenesis of HUVECs by tube formation assay. (J) Columnar statistics for tube formation. Scale bars, 100 μm. Data are represented as the means ± SD. *P < 0.05, **P < 0.01, ***P < 0.001, and ****P < 0.0001. ns, not significant.

First, we confirmed that HK-2 cells are epithelial cells using CK-19 fluorescence labeling (Fig. 2A). We investigated the functional role of USC-Exos in cell proliferation and migration. The EdU assay confirmed that USC-Exos remarkably enhanced the proliferation of HK-2 cells at increasing concentrations (Fig. 2B). The scratch and transwell assays confirmed that USC-Exos also promoted the migration of HK-2 cells at increasing concentrations (Fig. 2C and D). The results were statistically analyzed, and a statistical chart was drawn (Fig. 2E).

### USC-Exos promote proliferation, migration, and angiogenesis of HUVECs

To determine whether USC-Exos have a functional role in angiogenesis in vitro, we used Matrigel matrix culture medium to assess the effects of USC-Exos on the tube formation of HUVECs. The results demonstrated that USC-Exos remarkably enhanced tube formation at increasing concentrations compared to that of the group (0 μg/ml) in vitro (Fig. 2F). The EdU assay showed that USC-Exos remarkably enhanced the proliferation of HUVECs at increasing concentrations (Fig. 2G). The scratch and transwell assays showed that USC-Exos also promoted HUVEC migration at increasing concentrations (Fig. 2H and I). The above results were statistically significant, and the specific *P* value is indicated in the statistical chart (Fig. 2J and K).

### USC-Exos inhibit fibrosis and inflammation in HK-2 cells

**Fig. 3.**
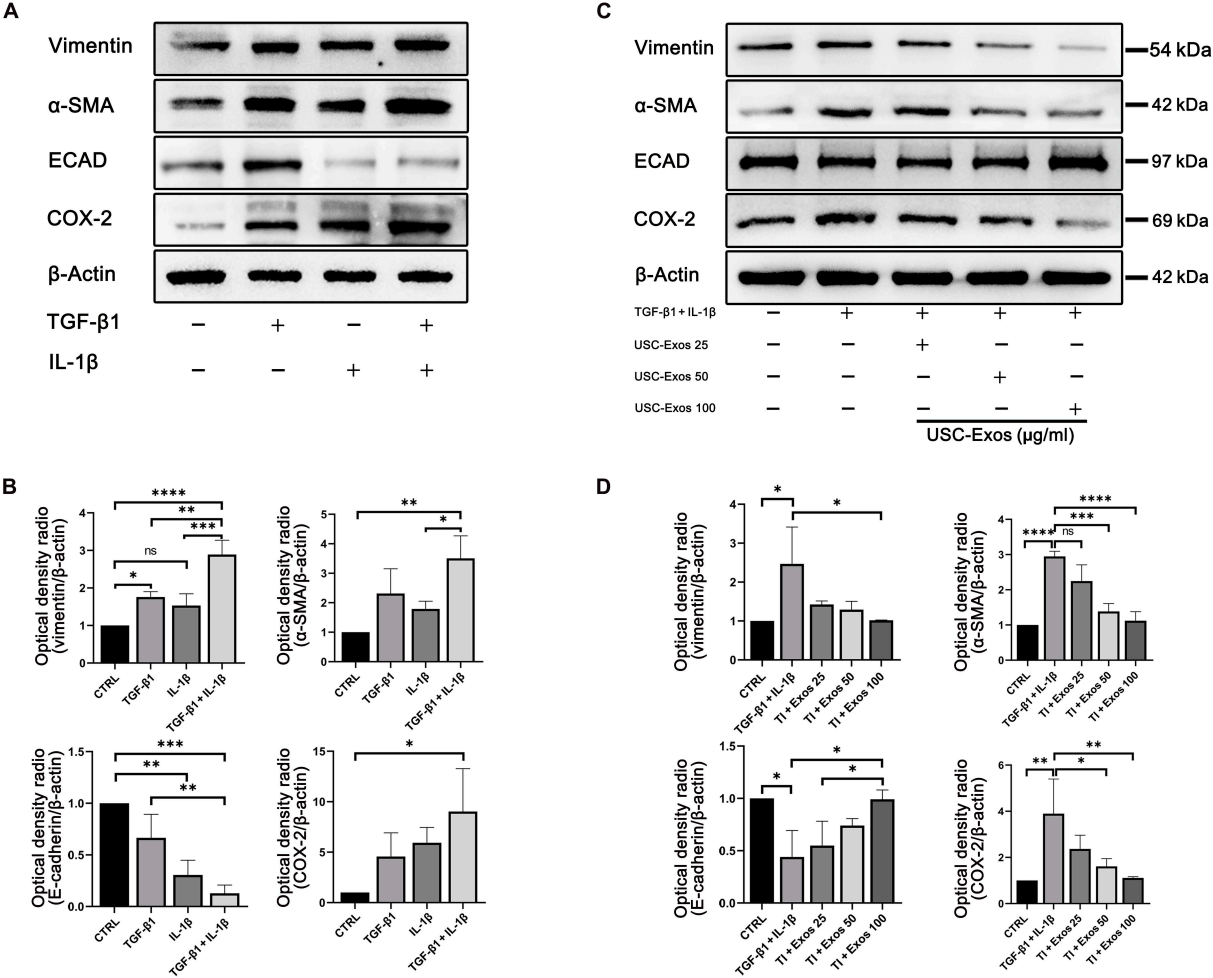
USC-Exos inhibited fibrosis and inflammation with increasing concentrations. (A and B) Western blot analysis of protein levels of vimentin, α-SMA, COX-2, and ECAD induced by TGF-β1 and/or IL-1β. (C and D) Western blot analysis of protein levels of vimentin, α-SMA, COX-2, and ECAD induced by TGF-β1 + IL-1β and different concentrations of USC-Exos. Data are represented as the means ± SD. *P < 0.05, **P < 0.01, ***P < 0.001, and ****P < 0.0001.

Then, we investigated whether USC-Exos affected fibrosis and inflammation in HK-2 cells. First, in vitro fibrosis and inflammation models were established by TGF-β1 and IL-1β. Western blot analysis showed that the combined use of TGF-β1 and IL-1β had a more negative effect than either alone (Fig. 3A and B). This finding is more consistent with the reality that 2 adverse factors always exist at the same time when tissue injury occurs. TGF-β1 and IL-1β remarkably increased the protein expression of COX-2, α-SMA, and vimentin and decreased the protein expression of ECAD. In subsequent experiments, Western blot analysis showed that USC-Exos protected against fibrosis and inflammation induced by TGF-β1 and IL-1β at increasing concentrations (Fig. 3C and D).

### miRNA sequencing and bioinformatics analysis

**Fig. 4.**
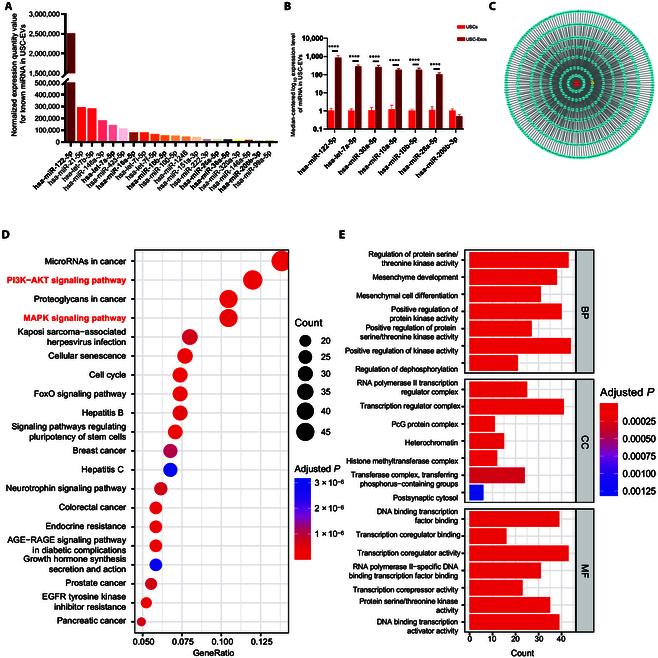
miRNA sequencing and bioinformatics analysis. (A) Expression levels of the top 20 miRNAs in USC-Exos. (B) Expression level of 7 potential therapeutic miRNAs in USC-Exos detected by qRT-PCR. (C) Target genes of miR-122-5p based on miRTarBase. (D and E) GO and KEGG functional enrichment analysis according to the *P* value from small to large, with the top 7 GO terms and the top 20 KEGG terms. EGFR, epidermal growth factor receptor. AGE-RAGE signaling pathway, advanced glycation end-products (AGEs)-receptor for AGEs (RAGE) signaling pathway; BP, biological process; CC, cellular component; MF, molecular function.

We screened out all miRNAs in USC-Exos through miRNA sequencing and sorted them according to the expression level. In this study, we selected the top 20 highly expressed miRNAs for display, which are shown in Fig. 4A. Among the top 20 highly expressed miRNAs, 7 miRNAs that play a therapeutic role in renal diseases were selected by consulting relevant literature with the name of each miRNA, renal diseases, nephropathy, renal fibrosis, and kidney injury as keywords. The miRNAs were as follows: miR-122-5p, miR-26a, miR-30, miR-10a, miR-10b, let-7a, and miR-200b. Related research is shown in Table S1. In addition, this study verified the expression of these 7 miRNAs with potential therapeutic effects in USC-Exos through qRT-PCR in vitro. The results were consistent with the results of high-throughput sequencing. The highest expression of miR-122-5p was found in USC-Exos, but miR-200b-3p with a high expression ranking in high-throughput sequencing was not found to have remarkably increased expression in this experiment (Fig. 4B). The relationship between miR-122-5p and its target genes was drawn by Cytoscape and is shown in Fig. 4C.

A functional enrichment analysis was carried out and included biological processes, cellular components, and molecular functions from the GO analysis and KEGG pathways. The biological processes were mainly enriched in “mesenchyme development” and “mesenchymal cell differentiation”, suggesting that USC exosomal miRNAs may play a positive role in the development and differentiation of mesenchymal stem cells. The main enriched cellular component was “RNA polymerase II transcription regulator complex”, and the main enriched molecular function was “DNA binding transcription factor binding”. The KEGG analysis suggested that the “PI3K-AKT signaling pathway” and “MAPK signaling pathway” were the main pathways (Fig. 4D and E). Consequently, we investigated whether USC-Exos play a role through these pathways in the following experiments.

### USC-Exos regulate the biological function of HK-2 cells via the PI3K-AKT and MAPK pathways

**Fig. 5.**
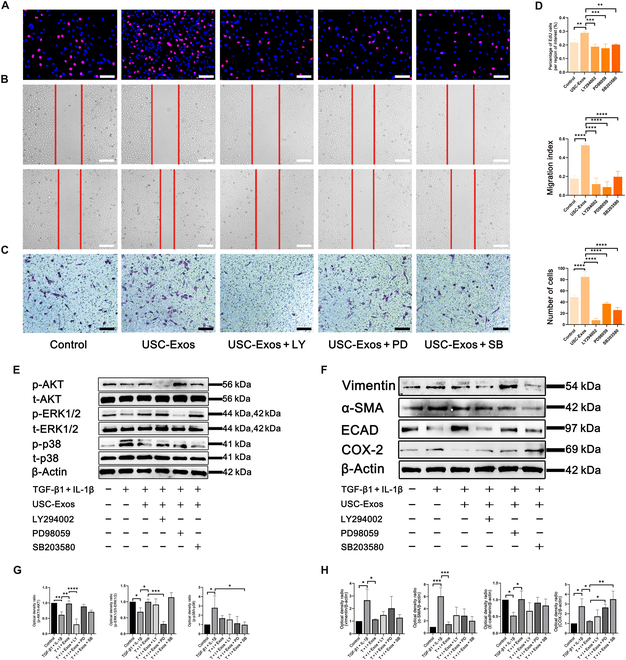
USC-Exos regulated the biological functions of HK-2 cells via the PI3K-AKT and MAPK pathways. (A) EdU assays showed that USC-Exo-mediated proliferation was suppressed by the inhibitors LY294002 (LY), PD98059 (PD), and SB203580 (SB). (B and C) Transwell and scratch assays showed that USC-Exo-mediated migration was suppressed by the inhibitors LY294002, PD98059, and SB203580. (D) Columnar statistics for proliferation and migration. (E and G) Western blot analysis of the protein levels of p-ERK1/2, p-p38, and p-AKT activated by USC-Exos and inhibited by TGF-β1 + IL-1β. LY294002, PD98059, and SB203580 inhibit the activation of AKT, ERK1/2, and p38 induced by USC-Exos, respectively. (F and H) Western blot analysis of protein levels of vimentin, α-SMA, and COX-2 inhibited by USC-Exos and promoted by TGF-β1 + IL-1β. The results of ECAD were the opposite; after the addition of inhibitors, inflammatory and fibrosis-related proteins all increased. Scale bars, 100 μm. Data are represented as the means ± SD. *P < 0.05, **P < 0.01, ***P < 0.001, and ****P < 0.0001.

On the basis of the results of KEGG enrichment analysis, the PI3K-AKT, MAPK-ERK1/2, and MAPK-p38 pathways were selected for verification**.** We pretreated HK-2 cells with LY294002, PD98059, and SB203580 for 30 min. Then, USC-Exos (100 μg/ml) were added for the EdU, scratch, and transwell assays. The ability of USC-Exos to promote cell proliferation and migration decreased remarkably with inhibition of these pathways (Fig. 5A to D). Western blot analysis showed that TGF-β1 and IL-1β decreased the expression of p-AKT and p-ERK1/2. USC-Exos increased the expression of p-AKT and p-ERK1/2 in HK-2 cells. This result indicated that USC-Exos activated the PI3K-AKT and MAPK-ERK1/2 signaling pathways after being absorbed by HK-2 cells. Interestingly, we found that both USC-Exos and TGF-β1 + IL-1β increased the expression of p-p38, while USC-Exos inhibited the overactivation of p-p38 induced by TGF-β1 and IL-1β. In addition, we pretreated HK-2 cells with the PI3K-AKT inhibitor LY294002, the MAPK-ERK1/2 inhibitor PD98059, and the MAPK-p38 inhibitor SB203580 for 30 min. Western blot analysis showed that LY294002, PD98059, and SB203580 inhibited the phosphorylation of AKT, ERK1/2, and p38, respectively (Fig. 5E and G).

We also investigated the protein expression of fibrosis and inflammation with inhibition of various pathways. The results showed that the fibrosis-related proteins α-SMA and vimentin were increased and ECAD was decreased upon inhibition of the PI3K-AKT and MAPK signaling pathways. The expression of the inflammation-related protein COX-2 was also remarkably increased with inhibition of the p38 and MAPK-ERK1/2 signaling pathways (Fig. 5F and H). When these pathways were blocked, the antifibrotic and anti-inflammatory effects of USC-Exos were remarkably reduced. Blocking the p38 signaling pathway has the most significant effect on inflammation, and blocking the PI3K-AKT signaling pathway has the most significant effect on fibrosis or epithelial interstitial transformation.

### USC-Exos promote renal tissue repair and inhibit fibrosis and inflammation in vivo

**Fig. 6.**
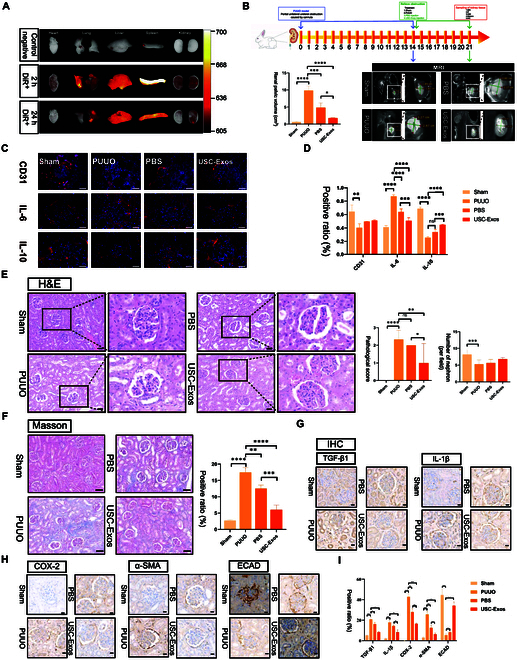
USC-Exos promoted renal tissue repair and inhibited fibrosis and inflammation in vivo. (A) DiR-labeled USC-Exo internalization by organs. (B) Animal experiment flow chart and representative MRI images of hydronephrosis of the sham, PUUO, PBS, and USC-Exo groups. Green lines and corresponding quantitative values depict the coronal length diameter of the left renal pelvis. IF, immunofluorescence; IHC, immunohistochemistry. (C) The expression of CD31^+^, IL-6^+^, and IL-10^+^ cells was detected by immunofluorescence. Scale bars, 50 μm. (D) Columnar statistics for the positive ratio of immunofluorescence. (E) The H&E staining of renal tissue (scale bars, 50 μm; the second role in H&E scale bars, 20 μm) and statistical analysis of pathological score and the number of nephrons. (F) Masson staining of renal tissue and the positive ratio of collagen fiber was analyzed. Scale bars, 50 μm. (G) The expression of TGF-β1 and IL-1β were detected by immunohistochemistry assays. Scale bars, 20 μm. (H) The expression of COX-2, α-SMA, and ECAD were detected by immunohistochemistry assays. Scale bars, 20 μm. (I) Quantitative analysis of fibrosis and inflammation-related factors. Data are represented as the means ± SD. *P < 0.05, **P < 0.01, ***P < 0.001, and ****P < 0.0001.

First, we determined the distribution of USC-Exos in vivo. DiR-labeled USC-Exos (400 μg/kg) were injected intravenously into experimental rabbits. Since rabbits are large experimental animals, organs, including the heart, lung, liver, spleen, and kidneys, were removed 2 and 24 h after the addition of DiR-labeled USC-Exos and imaged by an animal imaging system under the same conditions. Two hours after injection, the DiR-labeled USC-Exos were mainly distributed in the spleen, liver, and lungs, and a small amount was also distributed in the kidney. Twenty-four hours after injection, the tissue exchange and inactivation of USC-Exos in the spleen, liver, and lung ended, while the distribution and release of USC-Exos in the kidney were relatively stable (Fig. 6A).

The MRI results showed that the PBS group and USC-Exo group displayed less hydronephrosis than the PUUO group after relieving the obstruction. The effect of USC-Exos in promoting morphological recovery of the renal pelvis was more significant. The left RPV of the sham group was the lowest, followed by the USC-Exo group, whereas the PUUO group had the highest RPV. This finding is similar to the clinical situation. The renal pelvis retracts slightly by simply removing the obstruction, but the effect is not significant. In contrast, the USC-Exo group had better alleviation of hydronephrosis than the PBS group (Fig. 6B and Table 1). The ability of USC-Exos to promote the morphological recovery of the renal pelvis was significant.

We used anti-CD31 to label angiogenesis in vivo. The angiogenesis in the PUUO group decreased remarkably, and that in the PBS group and USC-Exo treatment group increased, among which the effect of the USC-Exo treatment group was greater (Fig. 6C and D). Compared with the PUUO group, the latter 2 groups showed an increasing trend, but there was no statistically significant difference.

In addition, we tested the representative indicators of inflammation through immunofluorescence. Through immunofluorescence, we found that the expression of IL-6 increased in the PUUO group, while the expression of IL-10 was remarkably decreased. IL-6 is a recognized proinflammatory factor, and IL-10 is an anti-inflammatory factor. However, USC-Exos inhibited the expression of IL-6 and promoted the expression of IL-10 compared to those of the PUUO group. Although the PBS group also had similar results, they were not as obvious as those in the USC-Exo group (Fig. 6C and D). These findings fully demonstrated the anti-inflammatory capacity of USC-Exos in vivo.

To further investigate whether USC-Exos attenuated renal damage and inhibited fibrosis and inflammation, we performed an in vivo study. There were significant pathological changes in the glomeruli and tubules in the PUUO group. H&E staining was used to assess the number of nephrons and the degree of damage to glomeruli and tubules. The results of the PUUO group showed that glomerular atrophy was obvious, many renal tubules were seriously dilated, the number of nephrons was remarkably reduced, the average injury area percentage was close to 50%, and the pathological score was the highest (Fig. 6E). Compared with the PUUO group, the PBS group and USC-Exo group showed improved glomerular morphology, degree of renal tubular expansion, pathological score, and number of nephrons. However, the pathological score and number of nephrons in the USC-Exo group were better than those in the PBS group. The number of nephrons is generally considered directly related to renal function. Masson staining showed that a large amount of collagen was deposited around the renal tubules in the PUUO group, suggesting that severe renal interstitial fibrosis occurred. Furthermore, the renal interstitial fibrosis area of the USC-Exo group was remarkably lower than that of the PUUO group (Fig. 6F).

In the immunohistochemistry experiment, we found that TGF-β1 and IL-1β were highly expressed in the PUUO group. USC-Exos inhibited PUUO-induced TGF-β1 and IL-1β production (Fig. 6G and I). The occurrence of renal interstitial fibrosis is largely due to epithelial–mesenchymal transformation. Therefore, we tested representative indicators of epithelial–mesenchymal transformation. The immunohistochemistry results showed that the expression of ECAD (renal tubular epithelial marker) decreased in the PUUO group, while the expression of α-SMA (fibroblast marker) was remarkably increased. Compared with the PUUO group, the USC-Exo group showed remarkably inhibited expression of α-SMA and enhanced expression of ECAD (Fig. 6H and I). These results suggested the potential role of USC-Exos in inhibiting renal interstitial fibrosis in UPJO. We also found that the expression of COX-2 was increased in the PUUO group. The activity of COX-2 in normal cells is very low. When cells are stimulated by inflammation, its expression level in inflammatory cells can rise to 10 to 80 times the normal level, resulting in an inflammatory response and tissue damage. USC-Exos also inhibited the expression of COX-2 after PUUO (Fig. 6H and I).

### Exosomal miR-122-5p promotes proliferation and migration of HK-2 cells

**Fig. 7.**
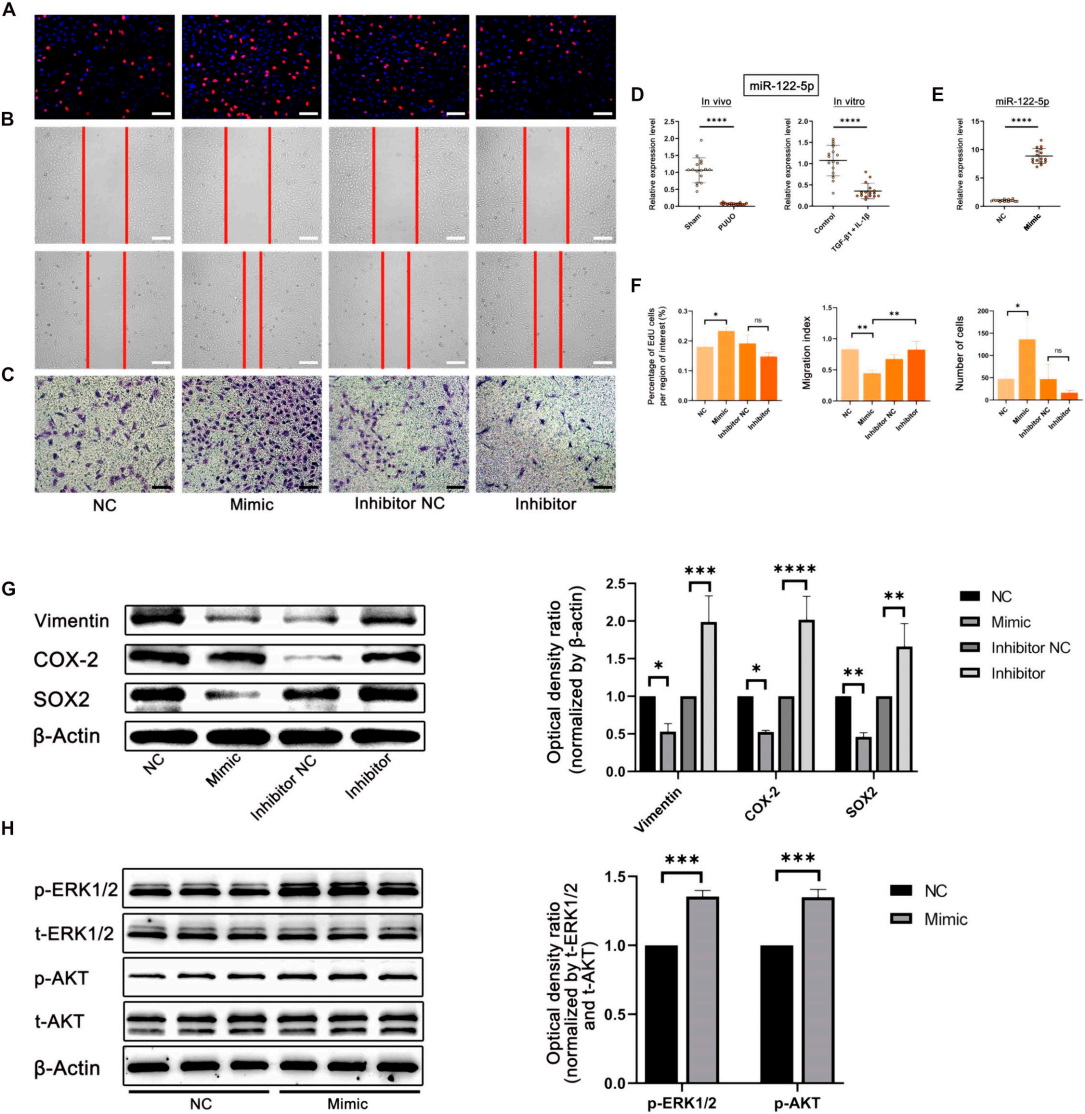
USC-Exos inhibited fibrosis and inflammation through exosomal miR-122-5p. (A to C) EdU, transwell, and scratch assays showed that miR-122-5p mimics promoted proliferation and migration. Scale bars, 100 μm. (D) Expression level of miR-122-5p in the sham in vitro/PUUO in vivo groups. (E) Columnar statistics for miR-122-5p when transfected with miR-122-5p NC/mimic. (F) Columnar statistics for proliferation and migration. SOX2 expression value in patients with CKD and renal tissue. (G) Western blot analysis of protein levels of vimentin, COX-2, and SOX2 after treatment by miR-122-5p mimics/inhibitors. (H) Western blot analysis of the protein levels of p-ERK1/2, t-ERK1/2, p-AKT, and t-AKT after treatment with miR-122-5p NC/mimic. Data are represented as the means ± SD. *P < 0.05, **P < 0.01, ***P < 0.001, and ****P < 0.0001.

According to our miRNA high-throughput sequencing and qRT-PCR analysis, miR-122-5p was the most highly expressed miRNA in USC-Exos. Through relevant literature, we found that miR-122-5p is a potential therapeutic miRNA in renal disease [29–32]. The EdU assay confirmed that miR-122-5p remarkably enhanced the proliferation of HK-2 cells, and when miR-122-5p was inhibited, cell proliferation remarkably decreased (Fig. 7A). The scratch and transwell assays confirmed that miR-122-5p also promoted the migration of HK-2 cells, and when miR-122-5p was inhibited, cell migration remarkably decreased (Fig. 7B and C). This study found that in the renal tissue of the PUUO modeling group and inflammation and fibrosis induced by TGF-β1 + IL-1β of HK-2, the expression level of miR-122-5p was remarkably lower than that of the sham group and control group, indicating the potential therapeutic effect of exogenous addition of miR-122-5p on damaged renal tissue (Fig. 7D). Therefore, we successfully transfected HK-2 cells with mimics to overexpress miR-122-5p (Fig. 7E). The results were statistically analyzed, and a statistical chart was drawn (Fig. 7F).

### Exosomal miR-122-5p inhibits fibrosis and inflammation and activates related pathways

Then, we investigated the protein expression of fibrosis and inflammation after transfection with miR-122-5p mimics. The results showed that the fibrosis-related protein vimentin was decreased in the miR-122-5p mimic group. The inflammation-related protein COX-2 was also decreased (Fig. 7G). After transfection with miR-122-5p mimics, the protein levels of p-AKT and p-ERK1/2 were remarkably increased. This result indicated that miR-122-5p mimics can also activate the PI3K-AKT and MAPK-ERK1/2 signaling pathways (Fig. 7H).

### SOX2 may be a negative regulatory factor in obstructive kidney injury

**Fig. 8.**
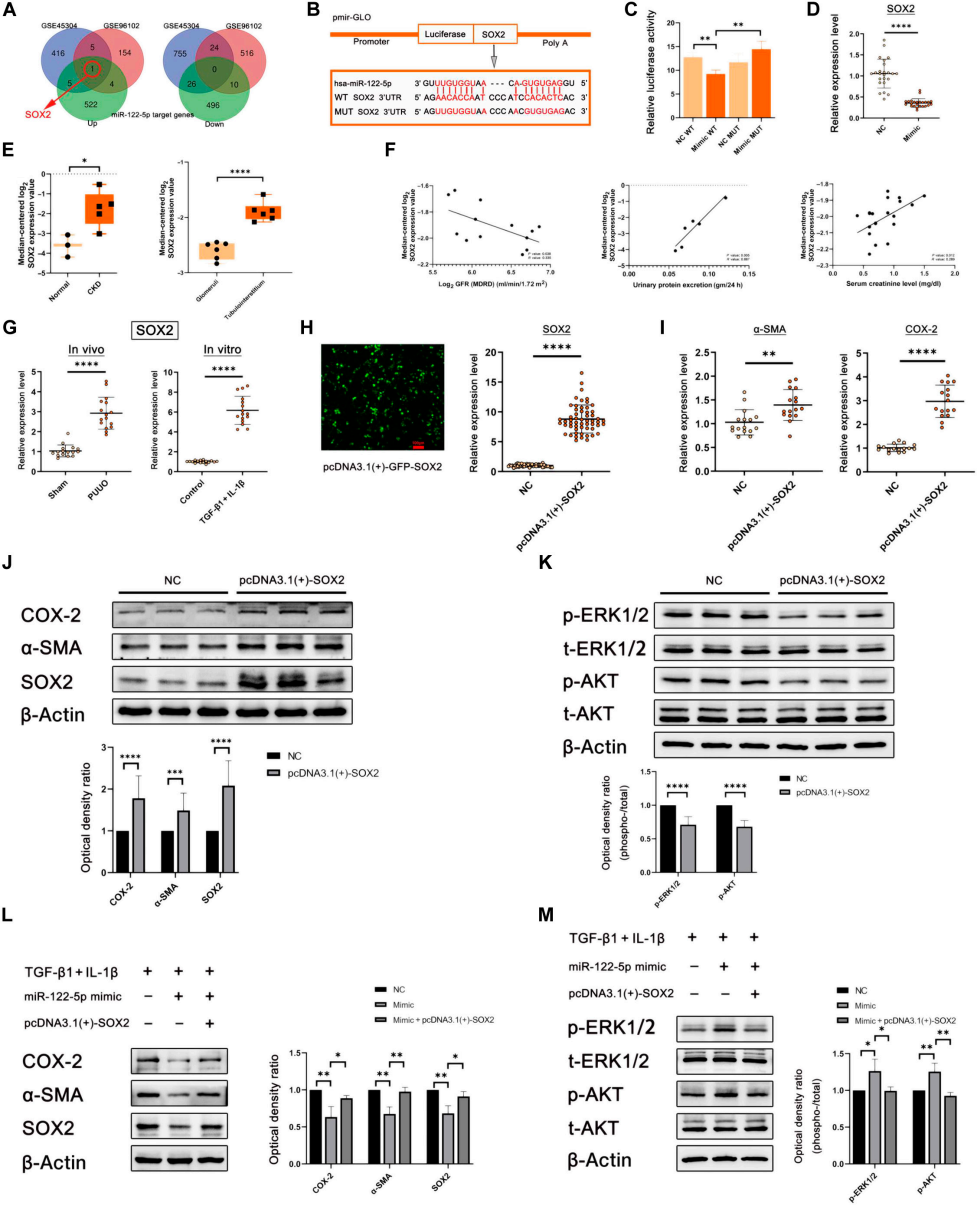
MiR-122-5p exerts anti-inflammatory and antifibrotic effects by targeting inhibition of SOX2 expression. (A) Venn diagram of the GSE45304, GSE96102, and miR-122-5p target genes. (B) Schematic diagram of dual luciferase reporter plasmid construction. WT, wild-type; MUT, mutant. (C) Columnar statistics for relative luciferase activity. (D) Expression level of SOX2 after treatment with miR-122-5p NC/mimic. (E) SOX2 expression value in patients with CKD and distribution in renal tissue. (F) The correlation between SOX2 and GFR/urinary protein extraction/serum creatinine. MDRD, modification of diet in renal disease. (G) Expression level of SOX2 in the sham in vitro/PUUO in vivo groups. (H) Transfection efficiency of pcDNA3.1(+)-GFP-SOX2 in fluorescence microscope and columnar statistics for SOX2 after treatment with NC/pcDNA3.1(+)-SOX2 by qRT-PCR. Scale bar, 100 μm. (I) Expression levels of COX-2 and α-SMA treated by NC/pcDNA3.1(+)-SOX2. (J) Western blot analysis of protein levels of COX-2, α-SMA, and SOX2 after treatment with NC/pcDNA3.1(+)-SOX2. (K) Western blot analysis of protein levels of p-ERK1/2 and p-AKT treated by NC/pcDNA3.1(+)-SOX2. (L) Western blot analysis of protein levels of COX-2, α-SMA, and SOX2 after treatment with TGF-β1 + IL-1β/miR-122-5p mimic/pcDNA3.1(+)-SOX2. (M) Western blot analysis of protein levels of p-ERK1/2 and p-AKT after treatment with TGF-β1 + IL-1β/miR-122-5p mimic/pcDNA3.1(+)-SOX2. Data are represented as the means ± SD. *P < 0.05, **P < 0.01, ***P < 0.001, and ****P < 0.0001.

To further determine the functions of miR-122-5p, we searched the miRNA target prediction analysis database MiRTarBase and found 532 genes as potential targets of miR-122-5p as described before. Then, we found 2 gene expression files (GSE45304 and GSE96102) from the GEO database that were related to PUUO [33,34]. GSE45304 contained 3 PUUO samples and 3 sham/normal samples. GSE96102 contained 36 PUUO samples and 39 sham/normal samples (Table S2). On the basis of *P* < 0.05 and |logFC| ≥ 0.3, a total of 1,232 DEGs were selected from GSE45304: 427 up-regulated genes and 805 down-regulated genes. In GSE96102, 714 DEGs were selected: 164 up-regulated genes and 550 down-regulated genes. All DEGs were identified by comparing PUUO samples with sham/normal samples. Then, we intersected their up-regulated DEGs and down-regulated DEGs in GSE45304 and GSE96102 and target genes of miR-122-5p separately. A Venn diagram was generated to show the intersection of the DEG profiles (Fig. 8A). SOX2 is an intersecting gene between up-regulated genes in PUUO and miR-122-5p target genes.

In addition, protein interactions among the DEGs of GSE45304 and GSE96102 were predicted with STRING tools. A total of 22 nodes and 28 edges were involved in the PPI network, as presented in Fig. S1. The top 10 genes evaluated by connectivity degree in the PPI network were identified (Table S3). Among the intersecting genes of GSE45304 and GSE96102, SOX2 has the highest degree, which means that the difference is the most significant (Fig. S1).

To verify the potential roles of SOX2 in renal disease, we conducted correlation analysis and subgroup analysis between SOX2 and clinical features using the Nephroseq v5 online tool. The results showed that the mRNA expression of SOX2 was positively correlated with renal disease. SOX2 was highly expressed in patients with CKD and had greater distribution in the tubulointerstitium than in the glomeruli (Fig. 8E). Thus, SOX2 may be a factor leading to renal tubulointerstitial fibrosis. In addition, SOX2 was negatively correlated with the GFR and positively correlated with serum creatinine and urine protein (Fig. 8F). This study found that in the renal tissue of the PUUO modeling group and inflammation and fibrosis induced by TGF-β1 + IL-1β of HK-2 cells, the expression level of SOX2 was remarkably higher than that of the sham group and control group, indicating the negative regulatory effect of SOX2 in obstructive kidney injury.

To further confirm this conclusion, we transfected the SOX2 overexpression plasmid into the HK-2 cell line. The transfection efficiency of overexpression was verified by transfection with green fluorescent protein (GFP)-labeled plasmids, qRT-PCR, and Western blotting (Fig. 8H and J). The results showed that SOX2 overexpression remarkably increased the expression of inflammation- and fibrosis-related proteins (Fig. 8J). The qRT-PCR results of COX-2 and α-SMA were consistent with the Western blot results (Fig. 8I). Overexpression of SOX2 remarkably decreased the expression of p-AKT and p-ERK1/2 (Fig. 8K).

### USC-Exos inhibit fibrosis and inflammation through exosomal miR-122-5p/SOX2

In addition to the evidence from bioinformatics analysis demonstrating the relationship between miR-122-5p and SOX2, we also found 3 predicted targets of miR-122-5p in the 3′UTR of the SOX2 transcript in miRTarBase. We selected a prediction target with the smallest minimum free energy. To verify the direct binding of miR-122-5p to the 3′UTR of the SOX2 gene, we then cloned the wild-type and mutant 3′UTR of SOX2 downstream of a firefly luciferase cassette in a luciferase reporter vector (Fig. 8B). Cotransfection of the miR-122-5p mimic with the wild-type reporter plasmids in human embryonic kidney 293T cells remarkably reduced the luciferase activity, and this effect was remarkably reversed by cotransfection with mutant reporter plasmids (Fig. 8C). These results indicated that miR-122-5p from USC-Exos may bind the SOX2 mRNA 3′UTR and thereby inhibit SOX2 expression via posttranslational repression. To further confirm the regulatory relationship between miR-122-5p and SOX2 in vitro, we transfected HK-2 cells with the miR-122-5p mimic. Then, decreased SOX2 expression was confirmed by qRT-PCR and Western blotting (Figs. 7G and 8D). Furthermore, Western blot analysis showed that cotransfection with the miR-122-5p mimic and SOX2 overexpression plasmid antagonized the anti-inflammatory and antifibrotic effects of miR-122-5p (Fig. 8L). We also tested the expression levels of p-AKT and p-ERK1/2 after cotransfection with the miR-122-5p mimic and SOX2 overexpression plasmid. Western blot analysis also showed that the miR-122-5p mimic could increase the protein expression of p-AKT and p-ERK1/2, and the addition of the SOX2 overexpression plasmid remarkably inhibited this effect (Fig. 8M). This result indicated that miR122-5p plays an anti-inflammatory and antifibrotic role and activated PI3K-AKT and MAPK signaling pathways by targeting SOX2.

## Discussion

UPJO is the most common cause of hydronephrosis [35] and the most common cause of obstructive kidney injury in children. Children with hydronephrosis often do not have specific clinical symptoms, which also leads to varying degrees of renal function damage and changes in renal fibrosis in some patients, especially older children. Even after the obstruction is relieved by surgery, the renal fibrosis and perioperative inflammation caused by long-term obstruction may still affect the renal function of the affected side. Therefore, after the surgical removal of the obstruction, additional intervention measures are needed to prevent the long-term effects of renal fibrosis and inflammation on the renal tissue. Research has shown that USCs play a positive role in tissue damage repair and have good effects on acute and chronic kidney injury diseases [36–38]. The tissue damage repair effect of USCs is mainly mediated by the paracrine release of USC-Exos. The effect of USC-Exos is mainly attributed to extracellular-derived miRNAs. To demonstrate whether USC-Exos inhibit fibrosis and inflammation caused by obstructive kidney injury and alleviate kidney injury, we performed in vitro and in vivo experiments, with the following results.

In vitro, USCs express pluripotent stem cell markers such as Nano G. USCs also express surface markers of mesenchymal stem cells, including CD73 and CD90, but do not express the hematopoietic stem cell markers, CD34 and CD45. The flow cytometry results showed that USCs expressed CD146, which is a podocyte marker. CD146 was expressed in parietal cells and podocytes of glomerular tissue and blood vessels of the human renal cortex but not in renal tubular epithelial cells or ureteral mucosa [39]. This study also investigated specific renal biomarkers, including WT-1 and nephrin. These positive renal biomarkers suggest a possible source of USCs.

USC-Exos were isolated from USC culture medium and identified as exosomes based on the expression of exosome-specific markers (such as TSG101, HSP70, and CD9) and the size of exosomes. PKH26 fluorescently labeled USC-Exos surround the target cells, confirming that USC-Exos can be effectively absorbed by the target cells. When ureteral obstruction occurs, renal parenchyma compression leads to reduced blood perfusion, and dilation of the collecting duct and distal tubules leads to interstitial fibrosis. By promoting the proliferation and migration of HK-2 and HUVECs, USC-Exos can effectively promote angiogenesis and reduce fibrosis. It was found that the higher the concentration of USC-Exos used, the stronger the effect. TGF-β1 and IL-1β were used to establish fibrosis and inflammation models in vitro and PUUO models in vivo, and the anti-inflammatory and antifibrotic effects of USC-Exos were validated at both the cellular and tissue levels.

This study aimed to determine the functional mechanism of USC-Exos. Therefore, we determined the type and expression of miRNAs in USC-Exos through high-throughput sequencing. We selected the top 20 miRNAs to predict their target genes and used these target genes for GO and KEGG enrichment analysis. We found that the main enriched biological processes were “mesenchymal development” and “mesenchymal differentiation”, indicating that USC-Exos and exosomal miRNAs may play a positive role in the development and differentiation of mesenchymal stem cells. KEGG enrichment analysis indicated that the main pathways included the PI3K-AKT and MAPK pathways. In subsequent pathway validation, this study found that USC-Exos can activate the PI3K-AKT and MAPK-ERK1/2 pathways and inhibit overactivation of the p38-MAPK pathway. In a study on acute lung injury induced by lipopolysaccharide, it was found that exosomal miR-150 can inhibit the excessive activation of phosphorylated p38 induced by lipopolysaccharide, thereby treating acute lung injury [40].

Although this study knows the downstream pathway of USC-Exos, it is still unclear which components in USC-Exos exert therapeutic effects. There are many components in exosomes, including proteins, bioactive lipids, and RNA. In particular, miRNA is abundant in exosomes and has been proven to be the main component and key molecule in exosomes. The role of exosomes may be the result of the synergistic effect of multiple miRNAs and their target genes. Through sequencing analysis, this study identified some miRNAs that are highly expressed in USC-Exos, some of which have a positive impact on the disease, while others have a negative impact. Therefore, this study reviewed previous relevant studies based on the obtained miRNA results. Through high-throughput sequencing, the most abundant miRNA was determined to be miR-122-5p. Among the top 20 highly expressed miRNAs, 7 may play therapeutic roles in kidney diseases, including miR-122-5p [29–32], miR-26a [41,42], miR-30 [43,44], miR-10a [45], miR-10b [45], let-7a [46], and miR-200b [47]. Some of their target genes may be involved in inhibiting renal fibrosis and promoting cell proliferation. Examples include pyruvate kinase M (PKM), TGFBR, zinc finger E-box binding homeobox 1 (ZEB1), and E2F Transcription Factor 2 (E2F2). Representative miRNAs with negative effects are also expressed in USC-Exos, such as the overexpression of miRNA-21-5p, which is believed to be closely related to end-stage renal disease with vascular calcification [48]. Therefore, although miRNA-21 is expressed in USC-Exos, it is a typical negative regulatory factor in kidney disease. Among the potential therapeutic exosomal miRNAs in this study, miR-122-5p was the most abundant miRNA. This finding was confirmed by high-throughput sequencing and qRT-PCR. In addition, this study found that the expression of miR-122-5p in renal tissue of the PUUO modeling group was remarkably lower than that of the sham group. In in vitro experiments, when inflammation and fibrosis were induced by TGF-β1 and IL-1β in renal tubular epithelial cells, the expression of miR-122-5p was also remarkably lower than that of the blank control group. Moreover, the role of miR-122-5p in kidney diseases and its ability to promote cell proliferation and migration have been reported [29–32]. In this study, miR-122-5p was also observed to promote the proliferation and migration of HK-2 cells and HUVECs. In in vitro experiments, after transfection with miR-122-5p mimics, the expression of inflammation- and fibrosis-related proteins remarkably decreased, while the expression of the pathway proteins phosphorylated AKT and phosphorylated ERK1/2 remarkably increased. This result suggests that the anti-inflammatory and antifibrotic effects of USC-Exos, as well as the activation of the PI3K-AKT and MAPK-ERK1/2 pathways, may be mainly achieved through miR-122-5p.

Therefore, this study further explored the downstream mechanism of miR-122-5p derived from USC-Exos and found that it can down-regulate the expression of SOX2. Previous studies have found that miR-122-5p can bind to SOX2, and miR-122-5p-mediated down-regulation of SOX2 is associated with cervical cancer [49]. SOX2 is a member of the SRY-related HMG box (SOX) family of transcription factors involved in regulating embryonic development and determining cell fate. The expression level of SOX2 is correlated with the degree of renal tubulointerstitial fibrosis and renal tubular cell damage [50,51]. In previous studies, it has been confirmed that SOX2 is highly expressed in patients with CKD and negatively correlated with renal function (such as GFR, creatinine/urea nitrogen, proteinuria, etc.) [52–54]. This study also confirmed that the expression of SOX2 in the kidney tissue of the PUUO modeling group was remarkably higher than that of the sham group. In in vitro experiments, when inflammation and fibrosis were induced by TGF-β1 and IL-1β in HK-2 cells, the expression of SOX2 was also remarkably increased. The possible negative regulatory role of SOX2 in obstructive kidney injury has been strongly supported. This study also verified that miR-122-5p can directly bind to the 3′UTR of SOX2 through a double luciferase reporter gene experiment. After transfection with the miR-122-5p mimic, the expression of SOX2 was also remarkably reduced. To verify the therapeutic effect of miR-122-5p targeting SOX2, we constructed a SOX2 overexpression plasmid. After overexpression of SOX2 in vitro, inflammation and fibrosis were remarkably enhanced, while the pathway proteins p-AKT and p-ERK1/2 were suppressed, indicating that SOX2 is positively correlated with inflammation and fibrosis and plays a negative regulatory role in obstructive kidney injury. When both the SOX2 overexpression plasmid and the miR-122-5p mimics were transfected simultaneously, the inhibitory effects of miR-122-5p on inflammation and fibrosis and activation of related pathways were both antagonized by SOX2. The mechanism of the miR-122-5p/SOX2 axis derived from USC-Exos in inhibiting inflammation and fibrosis after obstructive kidney injury discovered in this study may provide new molecular targets for the treatment of obstructive kidney injury.

Recent in vivo studies have described the therapeutic effects of USC-Exos on AKI and CKD. There is currently no report on the effect of USC-Exos on renal fibrosis and inflammation in UPJO postoperative hydronephrosis models. Thus, this experiment established a PUUO animal model representing the pathological changes of UPJO. The USC-Exos labeled with DiR can reach the kidneys and be absorbed by the affected kidney tissue, maintaining stability within 24 h. This study found that the CD31 fluorescence intensity of the PUUO group remarkably decreased and increased in the USC-Exo group. CD31 is mainly used to evaluate angiogenesis in tissues. This finding demonstrates the ability of USC-Exos to promote angiogenesis. IL-10 has the same fluorescence results as CD31. However, IL-6 showed the opposite result. IL-6 is a recognized proinflammatory factor, while IL-10 is an anti-inflammatory factor. From the pathological results, we found that the damage to the glomerulus and renal tubules after PUUO modeling is significant, and the number of nephrons is also remarkably reduced, with an increase in collagen deposition. Although there was some recovery after the obstruction was relieved, the effect was better in the USC-Exo group. The MRI results showed that the renal pelvis was remarkably dilated after PUUO modeling. Even if the obstruction was relieved, the shape of the renal pelvis was not effectively restored, and the intervention of USC-Exos remarkably promoted the restoration of renal pelvis morphology. The changes in inflammation and fibrotic indicators detected by immunohistochemistry were also consistent with the in vitro experiments. Especially after PUUO, the expression of TGF-β1 and IL-1β was increased, which is consistent with the use of TGF-β1 and IL-1β as a cell model in vitro. The expression of TGF-β1 and IL-1β in vivo was also inhibited by USC-Exos.

Limitation of this study is that it only analyzed and discussed the top 20 expressed miRNAs in USC-Exos. It is currently unclear whether there are poorly expressed but important miRNAs. In addition, this study did not experimentally validate all 7 miRNAs with potential therapeutic effects but only selected miR-122-5p with the highest expression level and therapeutic potential for validation. Moreover, the therapeutic effect of miR-122-5p was not verified in vivo. In subsequent studies, we will aim to validate these miRNAs one by one and explain which aspects of kidney disease are closely related to them.

## Conclusion

The results of this study indicate that in the PUUO model and TGF-β1 + IL-1β joint use in vitro and in vivo, USC-Exos can effectively internalize into HK-2 cells and HUVECs, promoting their proliferation, migration, and angiogenesis. USC-Exos reduce renal fibrosis and inflammation by activating the PI3K-AKT and MAPK pathways through the miR-122-5p/SOX2 axis derived from USC-Exos. These findings provide molecular therapeutic targets for patients with obstructive kidney injury.

## Ethical Approval

Ethics approval and consent to participate: This study involved human urine samples and animal experiments following the principles of the Helsinki Declaration. It was approved by the ethical committee of Harbin Medical University (file nos. SYDW2021-010 and KY2021-159). Consent for publication: Not applicable.

## Data Availability

The datasets used and/or analyzed during the current study are available from the corresponding author on reasonable request.
