# Supplementary material for: The Inhibition of Fibrosis and Inflammation in Obstructive Kidney Injury via the miR-122-5p/SOX2 Axis Using USC-Exos
Source: Biomater Res. 2024 Apr 10;28:0013. doi: 10.34133/bmr.0013 (PMC11014086; doi:10.34133/bmr.0013)
Supplement: Supplementary 1 — Fig. S1 Tables S1 to S4 [file bmr.0013.f1.zip › Supplementary Table 1.docx]

Supplementary Table 1. Potential therapeutic miRNAs involved in related renal diseases.

| miRNA involved | First author, Year | Disease model | Effect |
| --- | --- | --- | --- |
| miR-122-5p | Hu, 2018[1] | Uric acid nephropathy (UAN) in mouse | LncRNA ANRIL promotes NLRP3 inflammasome activation through miR-122-5p/BRCC3 axis |
|  | Liu, 2022[2] | UUO in mouse | MiR-122-5p expression was down-regulated and PKM expression was up-regulated in HK2 cells treated with TGF-β1. Radix Rehmanniae (RR) was able to promote the up-regulation of miR-122-5p expression in UUO mice in vivo. RR inhibited renal fibrosis progression by regulating the miR-122-5p/PKM axis. |
|  | Wang, 2019[3] | Renal carcinoma | Overexpression of mir-122-5p promotes the survival, proliferation, migration, glycolysis and autophagy of renal cell carcinoma by negatively regulating PKM2 |
| miR-26a | Ichii, 2014[4] | Autoimmune glomerulonephritis in mouse | MiR-26a regulates podocyte differentiation and cytoskeletal integrity, and its altered levels in glomerulus and urine may serve as a marker of injured podocytes in autoimmune glomerulonephritis. |
|  | Jansen, 2016[5] | Diabetic patient vs normal control | MiR-26a were  significantly reduced in diabetic patients compared to non-diabetic patients. Patients with low miR-26a levels were at higher risk for a concomitant coronary artery disease. |
| miR-26a&miR-30c | Zheng, 2016[6] | Spontaneous type 2 diabetes in mouse | Overexpression of miR-26a and miR-30c coordinately decreased CTGF protein levels and subsequently ameliorated TGFβ1 induced EMT in NRK-52E cells. |
| miR-26a&miR-10a/b | Ichii, 2017[7] | CKD model in dog | Urinary exosome derived miR-26a and miR-10a/b was significantly decreased in CKD dogs, and the decrease of miR-26a and miR-10a/b in the glomerulus and miR-10b in the tubulointerstitium negatively correlated with deteriorated renal function and histopathology. |
| let-7a | Tangtanatakul, 2019[8] | Active lupus nephritis (LN) compared to inactive LN | Let-7a was significantly down-regulated in LN patients with active disease compared with inactive disease. |
| miR-30 | Gu, 2016[9] | IRI in mouse | Human Wharton Jelly mesenchymal stromal cells derived extracellular vesicles could protect the kidney  from IRI by inhibition of mitochondrial fission via miR-30. |
| miR-200b | Yu, 2018[10] | CKD patient vs normal control | MiR-200b was lower in the CKD group than in the normal group and decreased more  significantly with fibrosis progression as well as in IgA nephropathy and diabetic kidney disease. |

**References**

1. Hu J, Wu H, Wang D, Yang Z, Dong J: **LncRNA ANRIL promotes NLRP3 inflammasome activation in uric acid nephropathy through miR-122-5p/BRCC3 axis**. *Biochimie* 2019, **157**:102-110.

2. Liu X, Xu H, Zang Y, Liu W, Sun X: **Radix Rehmannia Glutinosa inhibits the development of renal fibrosis by regulating miR-122-5p/PKM axis**. *Am J Transl Res* 2022, **14**:103-119.

3. Wang S, Zheng W, Ji A, Zhang D, Zhou M: **Overexpressed miR-122-5p Promotes Cell Viability, Proliferation, Migration And Glycolysis Of Renal Cancer By Negatively Regulating PKM2**. *Cancer Manag Res* 2019, **11**:9701-9713.

4. Ichii O, Otsuka-Kanazawa S, Horino T, Kimura J, Nakamura T, Matsumoto M, Toi M, Kon Y: **Decreased miR-26a expression correlates with the progression of podocyte injury in autoimmune glomerulonephritis**. *PLoS One* 2014, **9**(10):e110383.

5. Jansen F, Wang H, Przybilla D, Franklin BS, Dolf A, Pfeifer P, Schmitz T, Flender A, Endl E, Nickenig G *et al*: **Vascular endothelial microparticles-incorporated microRNAs are altered in patients with diabetes mellitus**. *Cardiovasc Diabetol* 2016, **15**:49.

6. Zheng Z, Guan M, Jia Y, Wang D, Pang R, Lv F, Xiao Z, Wang L, Zhang H, Xue Y: **The coordinated roles of miR-26a and miR-30c in regulating TGFbeta1-induced epithelial-to-mesenchymal transition in diabetic nephropathy**. *Sci Rep* 2016, **6**:37492.

7. Ichii O, Ohta H, Horino T, Nakamura T, Hosotani M, Mizoguchi T, Morishita K, Nakamura K, Hoshino Y, Takagi S *et al*: **Urinary exosome-derived microRNAs reflecting the changes of renal function and histopathology in dogs**. *Sci Rep* 2017, **7**:40340.

8. Tangtanatakul P, Klinchanhom S, Sodsai P, Sutichet T, Promjeen C, Avihingsanon Y, Hirankarn N: **Down-regulation of let-7a and miR-21 in urine exosomes from lupus nephritis patients during disease flare**. *Asian Pac J Allergy Immunol* 2019, **37**(4):189-197.

9. Gu D, Zou X, Ju G, Zhang G, Bao E, Zhu Y: **Mesenchymal Stromal Cells Derived Extracellular Vesicles Ameliorate Acute Renal Ischemia Reperfusion Injury by Inhibition of Mitochondrial Fission through miR-30**. *Stem Cells Int* 2016, **2016**:2093940.

10. Yu Y, Bai F, Qin N, Liu W, Sun Q, Zhou Y, Yang J: **Non-Proximal Renal Tubule-Derived Urinary Exosomal miR-200b as a Biomarker of Renal Fibrosis**. *Nephron* 2018, **139**(3):269-282.
