# Supplementary material for: The Inhibition of Fibrosis and Inflammation in Obstructive Kidney Injury via the miR-122-5p/SOX2 Axis Using USC-Exos
Source: Biomater Res. 2024 Apr 10;28:0013. doi: 10.34133/bmr.0013 (PMC11014086; doi:10.34133/bmr.0013)
Supplement: Supplementary 1 — Fig. S1 Tables S1 to S4 [file bmr.0013.f1.zip › Supplementary Table 2.docx]

**Supplementary Table 2.** Statistics of partial unilateral ureteral obstruction derived from the GEO database.

| **Dataset ID** | **PUUO** | **Sham/Normal** | **Total number** |
| --- | --- | --- | --- |
| GSE45304 | 3 | 3 | 6 |
| GSE96102 | 36 | 39 | 75 |
