# Supplementary material for: The Inhibition of Fibrosis and Inflammation in Obstructive Kidney Injury via the miR-122-5p/SOX2 Axis Using USC-Exos
Source: Biomater Res. 2024 Apr 10;28:0013. doi: 10.34133/bmr.0013 (PMC11014086; doi:10.34133/bmr.0013)
Supplement: Supplementary 1 — Fig. S1 Tables S1 to S4 [file bmr.0013.f1.zip › Supplementary Table 3.docx]

**Supplementary Table 3.** Top 10 hub genes with higher degree of connectivity in PUUO vs Sham/Normal DEGs.

| **Gene symbol** | **Gene description** | **Degree** |
| --- | --- | --- |
| SOX2 | (Sex determining region Y)-box 2 | 7 |
| HIST1H2AF | Not found | 5 |
| FGF2 | Fibroblast growth factor 2 | 5 |
| BUB1B | BUB1 mitotic checkpoint serine/threonine kinase B | 4 |
| TRIM52 | Tripartite motif containing 52 | 3 |
| GNAS | GNAS complex locus | 3 |
| MUC1 | Mucin 1, cell surface associated | 3 |
| CPE | Carboxypeptidase E | 3 |
| CKS2 | CDC28 protein kinase regulatory subunit 2 | 3 |
| RBBP8 | Retinoblastoma binding protein 8 | 3 |
