# Supplementary material for: The Inhibition of Fibrosis and Inflammation in Obstructive Kidney Injury via the miR-122-5p/SOX2 Axis Using USC-Exos
Source: Biomater Res. 2024 Apr 10;28:0013. doi: 10.34133/bmr.0013 (PMC11014086; doi:10.34133/bmr.0013)
Supplement: Supplementary 1 — Fig. S1 Tables S1 to S4 [file bmr.0013.f1.zip › Supplementary Table 4.docx]

**Supplementary Table 4.** Target genes of miR-122-5p based on miRTarBase database.

| miRNA | Target gene |
| --- | --- |
| hsa-miR-122-5p | CYP7A1 |
| hsa-miR-122-5p | WNT1 |
| hsa-miR-122-5p | IGF1R |
| hsa-miR-122-5p | SRF |
| hsa-miR-122-5p | RAC1 |
| hsa-miR-122-5p | RHOA |
| hsa-miR-122-5p | PRKRA |
| hsa-miR-122-5p | CCNG1 |
| hsa-miR-122-5p | GTF2B |
| hsa-miR-122-5p | GYS1 |
| hsa-miR-122-5p | ANK2 |
| hsa-miR-122-5p | NFATC2IP |
| hsa-miR-122-5p | ENTPD4 |
| hsa-miR-122-5p | ANXA11 |
| hsa-miR-122-5p | ALDOA |
| hsa-miR-122-5p | RAB6B |
| hsa-miR-122-5p | RAB11FIP1 |
| hsa-miR-122-5p | FOXP1 |
| hsa-miR-122-5p | MECP2 |
| hsa-miR-122-5p | NCAM1 |
| hsa-miR-122-5p | UBAP2 |
| hsa-miR-122-5p | TBX19 |
| hsa-miR-122-5p | AACS |
| hsa-miR-122-5p | DUSP2 |
| hsa-miR-122-5p | ATP1A2 |
| hsa-miR-122-5p | MAPK11 |
| hsa-miR-122-5p | FUNDC2 |
| hsa-miR-122-5p | AKT3 |
| hsa-miR-122-5p | TPD52L2 |
| hsa-miR-122-5p | GALNT10 |
| hsa-miR-122-5p | G6PC3 |
| hsa-miR-122-5p | AP3M2 |
| hsa-miR-122-5p | SLC7A1 |
| hsa-miR-122-5p | XPO6 |
| hsa-miR-122-5p | FOXJ3 |
| hsa-miR-122-5p | SLC7A11 |
| hsa-miR-122-5p | TRIB1 |
| hsa-miR-122-5p | EGLN3 |
| hsa-miR-122-5p | NUMBL |
| hsa-miR-122-5p | ADAM17 |
| hsa-miR-122-5p | DSTYK |
| hsa-miR-122-5p | FAM117B |
| hsa-miR-122-5p | BCL2L2 |
| hsa-miR-122-5p | PRKAB1 |
| hsa-miR-122-5p | ADAM10 |
| hsa-miR-122-5p | ACVR1C |
| hsa-miR-122-5p | PTPN1 |
| hsa-miR-122-5p | NT5C3A |
| hsa-miR-122-5p | P4HA1 |
| hsa-miR-122-5p | ZNF395 |
| hsa-miR-122-5p | SOCS1 |
| hsa-miR-122-5p | SSR3 |
| hsa-miR-122-5p | PHOX2A |
| hsa-miR-122-5p | DZIP1L |
| hsa-miR-122-5p | GSTM2 |
| hsa-miR-122-5p | RAI14 |
| hsa-miR-122-5p | STARD13 |
| hsa-miR-122-5p | CNN3 |
| hsa-miR-122-5p | A2M |
| hsa-miR-122-5p | EFCAB6 |
| hsa-miR-122-5p | GLOD4 |
| hsa-miR-122-5p | CALR |
| hsa-miR-122-5p | POFUT1 |
| hsa-miR-122-5p | PYGO1 |
| hsa-miR-122-5p | CDY2B |
| hsa-miR-122-5p | CDC42EP3 |
| hsa-miR-122-5p | CS |
| hsa-miR-122-5p | RNF170 |
| hsa-miR-122-5p | GTF3C6 |
| hsa-miR-122-5p | ZNF321P |
| hsa-miR-122-5p | LAMP1 |
| hsa-miR-122-5p | SLC52A2 |
| hsa-miR-122-5p | ZNF658 |
| hsa-miR-122-5p | ATP13A3 |
| hsa-miR-122-5p | CPNE5 |
| hsa-miR-122-5p | KATNAL1 |
| hsa-miR-122-5p | TMEM136 |
| hsa-miR-122-5p | ATP11A |
| hsa-miR-122-5p | TMEM74 |
| hsa-miR-122-5p | ANKRD10 |
| hsa-miR-122-5p | TBL1XR1 |
| hsa-miR-122-5p | USP10 |
| hsa-miR-122-5p | AKAP11 |
| hsa-miR-122-5p | BACH2 |
| hsa-miR-122-5p | HCCS |
| hsa-miR-122-5p | GTDC2 |
| hsa-miR-122-5p | CTPS1 |
| hsa-miR-122-5p | SLC44A1 |
| hsa-miR-122-5p | CLSPN |
| hsa-miR-122-5p | RABGEF1 |
| hsa-miR-122-5p | FBXO7 |
| hsa-miR-122-5p | NCDN |
| hsa-miR-122-5p | SOX2 |
| hsa-miR-122-5p | ZNF618 |
| hsa-miR-122-5p | ABLIM1 |
| hsa-miR-122-5p | ZBTB4 |
| hsa-miR-122-5p | LUZP1 |
| hsa-miR-122-5p | NLGN3 |
| hsa-miR-122-5p | PRR11 |
| hsa-miR-122-5p | HHAT |
| hsa-miR-122-5p | UBE2L3 |
| hsa-miR-122-5p | DNAJC18 |
| hsa-miR-122-5p | FAM102A |
| hsa-miR-122-5p | PFKFB2 |
| hsa-miR-122-5p | DENND2C |
| hsa-miR-122-5p | HMOX1 |
| hsa-miR-122-5p | USP28 |
| hsa-miR-122-5p | KRT18 |
| hsa-miR-122-5p | BPGM |
| hsa-miR-122-5p | ZNF233 |
| hsa-miR-122-5p | SLC25A30 |
| hsa-miR-122-5p | ANKRD9 |
| hsa-miR-122-5p | RLN1 |
| hsa-miR-122-5p | DDIT3 |
| hsa-miR-122-5p | TRIM65 |
| hsa-miR-122-5p | B4GALT1 |
| hsa-miR-122-5p | ANXA7 |
| hsa-miR-122-5p | SLC19A2 |
| hsa-miR-122-5p | MARCKS |
| hsa-miR-122-5p | CLEC11A |
| hsa-miR-122-5p | CENPF |
| hsa-miR-122-5p | CLDN18 |
| hsa-miR-122-5p | PFDN1 |
| hsa-miR-122-5p | SPAG9 |
| hsa-miR-122-5p | CEACAM8 |
| hsa-miR-122-5p | KRT10 |
| hsa-miR-122-5p | SET |
| hsa-miR-122-5p | SLC11A2 |
| hsa-miR-122-5p | MYCBP |
| hsa-miR-122-5p | GPHB5 |
| hsa-miR-122-5p | STAU2 |
| hsa-miR-122-5p | NFATC1 |
| hsa-miR-122-5p | ERP29 |
| hsa-miR-122-5p | MEP1A |
| hsa-miR-122-5p | SPTLC1 |
| hsa-miR-122-5p | GTF2H2 |
| hsa-miR-122-5p | C14orf39 |
| hsa-miR-122-5p | UBE2K |
| hsa-miR-122-5p | PPP1R9B |
| hsa-miR-122-5p | PSMD10 |
| hsa-miR-122-5p | CALD1 |
| hsa-miR-122-5p | TTYH3 |
| hsa-miR-122-5p | NMNAT2 |
| hsa-miR-122-5p | SCN4B |
| hsa-miR-122-5p | C1orf122 |
| hsa-miR-122-5p | PAK1 |
| hsa-miR-122-5p | DNAJB1 |
| hsa-miR-122-5p | DMXL1 |
| hsa-miR-122-5p | ZNF160 |
| hsa-miR-122-5p | CSRP1 |
| hsa-miR-122-5p | PHKA1 |
| hsa-miR-122-5p | RBBP5 |
| hsa-miR-122-5p | ANKRD13C |
| hsa-miR-122-5p | SUCLA2 |
| hsa-miR-122-5p | PEA15 |
| hsa-miR-122-5p | MTPN |
| hsa-miR-122-5p | YKT6 |
| hsa-miR-122-5p | MOB3B |
| hsa-miR-122-5p | FAM118A |
| hsa-miR-122-5p | ZNF264 |
| hsa-miR-122-5p | HECTD3 |
| hsa-miR-122-5p | BATF2 |
| hsa-miR-122-5p | PIP4K2A |
| hsa-miR-122-5p | MAPRE1 |
| hsa-miR-122-5p | TBC1D22B |
| hsa-miR-122-5p | SLC9A1 |
| hsa-miR-122-5p | NPEPPS |
| hsa-miR-122-5p | BCL2L1 |
| hsa-miR-122-5p | OSMR |
| hsa-miR-122-5p | CALU |
| hsa-miR-122-5p | BRI3BP |
| hsa-miR-122-5p | PSPH |
| hsa-miR-122-5p | APMAP |
| hsa-miR-122-5p | WASF1 |
| hsa-miR-122-5p | LCA5 |
| hsa-miR-122-5p | NODAL |
| hsa-miR-122-5p | CASP7 |
| hsa-miR-122-5p | CPA3 |
| hsa-miR-122-5p | PALM |
| hsa-miR-122-5p | TCP11 |
| hsa-miR-122-5p | NALCN |
| hsa-miR-122-5p | PLAGL2 |
| hsa-miR-122-5p | IDS |
| hsa-miR-122-5p | PARP11 |
| hsa-miR-122-5p | MAZ |
| hsa-miR-122-5p | CPNE4 |
| hsa-miR-122-5p | FAM19A3 |
| hsa-miR-122-5p | KRT14 |
| hsa-miR-122-5p | DYNC1H1 |
| hsa-miR-122-5p | GNL3L |
| hsa-miR-122-5p | HLA-DQA1 |
| hsa-miR-122-5p | EYA4 |
| hsa-miR-122-5p | GNPDA2 |
| hsa-miR-122-5p | BRCA2 |
| hsa-miR-122-5p | ZSCAN4 |
| hsa-miR-122-5p | HSPA5 |
| hsa-miR-122-5p | SERAC1 |
| hsa-miR-122-5p | SLC15A2 |
| hsa-miR-122-5p | RABIF |
| hsa-miR-122-5p | ART3 |
| hsa-miR-122-5p | EP400 |
| hsa-miR-122-5p | MT4 |
| hsa-miR-122-5p | TRAM2 |
| hsa-miR-122-5p | PHPT1 |
| hsa-miR-122-5p | KIAA0101 |
| hsa-miR-122-5p | VHL |
| hsa-miR-122-5p | IFNA1 |
| hsa-miR-122-5p | FSTL3 |
| hsa-miR-122-5p | PHF14 |
| hsa-miR-122-5p | ZCCHC2 |
| hsa-miR-122-5p | GSTM3 |
| hsa-miR-122-5p | DCTN5 |
| hsa-miR-122-5p | CHST3 |
| hsa-miR-122-5p | HECW2 |
| hsa-miR-122-5p | ADO |
| hsa-miR-122-5p | POMZP3 |
| hsa-miR-122-5p | CHST12 |
| hsa-miR-122-5p | ARSB |
| hsa-miR-122-5p | ATP7A |
| hsa-miR-122-5p | PMP22 |
| hsa-miR-122-5p | TGFBRAP1 |
| hsa-miR-122-5p | ORC2 |
| hsa-miR-122-5p | CREB1 |
| hsa-miR-122-5p | CD83 |
| hsa-miR-122-5p | TOB2 |
| hsa-miR-122-5p | LRP11 |
| hsa-miR-122-5p | MPV17 |
| hsa-miR-122-5p | TRIM29 |
| hsa-miR-122-5p | OSBP2 |
| hsa-miR-122-5p | PKM |
| hsa-miR-122-5p | FOXK2 |
| hsa-miR-122-5p | CLIC4 |
| hsa-miR-122-5p | ST6GALNAC4 |
| hsa-miR-122-5p | SMURF2 |
| hsa-miR-122-5p | LMNB2 |
| hsa-miR-122-5p | BAX |
| hsa-miR-122-5p | CDK4 |
| hsa-miR-122-5p | Cux1 |
| hsa-miR-122-5p | TNRC6A |
| hsa-miR-122-5p | VMA21 |
| hsa-miR-122-5p | ACER2 |
| hsa-miR-122-5p | HIATL1 |
| hsa-miR-122-5p | MEF2D |
| hsa-miR-122-5p | TGFB1 |
| hsa-miR-122-5p | AXL |
| hsa-miR-122-5p | NOD2 |
| hsa-miR-122-5p | FUT8 |
| hsa-miR-122-5p | CYP3A5 |
| hsa-miR-122-5p | OLR1 |
| hsa-miR-122-5p | EFTUD2 |
| hsa-miR-122-5p | MRPL52 |
| hsa-miR-122-5p | OSBPL10 |
| hsa-miR-122-5p | CDKL1 |
| hsa-miR-122-5p | ZSWIM1 |
| hsa-miR-122-5p | PIGO |
| hsa-miR-122-5p | G6PC |
| hsa-miR-122-5p | TNFSF14 |
| hsa-miR-122-5p | WSB1 |
| hsa-miR-122-5p | SUMO1 |
| hsa-miR-122-5p | REL |
| hsa-miR-122-5p | RAD21 |
| hsa-miR-122-5p | MDM4 |
| hsa-miR-122-5p | MAFK |
| hsa-miR-122-5p | FHL2 |
| hsa-miR-122-5p | FBXO21 |
| hsa-miR-122-5p | CCDC43 |
| hsa-miR-122-5p | UBTF |
| hsa-miR-122-5p | LRP3 |
| hsa-miR-122-5p | PRMT3 |
| hsa-miR-122-5p | ZNF485 |
| hsa-miR-122-5p | CIRH1A |
| hsa-miR-122-5p | ABCF2 |
| hsa-miR-122-5p | SLC7A5 |
| hsa-miR-122-5p | ZNF354B |
| hsa-miR-122-5p | TOMM70A |
| hsa-miR-122-5p | SLC2A3 |
| hsa-miR-122-5p | GTF2F1 |
| hsa-miR-122-5p | COPA |
| hsa-miR-122-5p | NCAPD2 |
| hsa-miR-122-5p | MCAM |
| hsa-miR-122-5p | GLUL |
| hsa-miR-122-5p | FBXO27 |
| hsa-miR-122-5p | CDK19 |
| hsa-miR-122-5p | BIRC5 |
| hsa-miR-122-5p | YWHAB |
| hsa-miR-122-5p | SIGLEC12 |
| hsa-miR-122-5p | C19orf52 |
| hsa-miR-122-5p | SLC4A1 |
| hsa-miR-122-5p | PATZ1 |
| hsa-miR-122-5p | YIPF4 |
| hsa-miR-122-5p | RBM47 |
| hsa-miR-122-5p | PARVB |
| hsa-miR-122-5p | HSPA4L |
| hsa-miR-122-5p | GALNT3 |
| hsa-miR-122-5p | ABCF1 |
| hsa-miR-122-5p | RBM43 |
| hsa-miR-122-5p | ERC1 |
| hsa-miR-122-5p | AP1S1 |
| hsa-miR-122-5p | HDDC2 |
| hsa-miR-122-5p | ORC6 |
| hsa-miR-122-5p | PPIC |
| hsa-miR-122-5p | ZNF322P1 |
| hsa-miR-122-5p | PKNOX1 |
| hsa-miR-122-5p | PRSS16 |
| hsa-miR-122-5p | HM13 |
| hsa-miR-122-5p | POM121L7 |
| hsa-miR-122-5p | HEBP2 |
| hsa-miR-122-5p | ABL2 |
| hsa-miR-122-5p | BTN3A2 |
| hsa-miR-122-5p | RHBDL2 |
| hsa-miR-122-5p | PHLDA3 |
| hsa-miR-122-5p | ANG |
| hsa-miR-122-5p | SNX22 |
| hsa-miR-122-5p | ZFP14 |
| hsa-miR-122-5p | SYAP1 |
| hsa-miR-122-5p | RABL3 |
| hsa-miR-122-5p | CCS |
| hsa-miR-122-5p | MTO1 |
| hsa-miR-122-5p | HLA-E |
| hsa-miR-122-5p | MEAF6 |
| hsa-miR-122-5p | TIAL1 |
| hsa-miR-122-5p | YME1L1 |
| hsa-miR-122-5p | XKR6 |
| hsa-miR-122-5p | WBSCR27 |
| hsa-miR-122-5p | ORAI2 |
| hsa-miR-122-5p | NAA50 |
| hsa-miR-122-5p | DGKE |
| hsa-miR-122-5p | AGAP9 |
| hsa-miR-122-5p | AKR7L |
| hsa-miR-122-5p | OPTN |
| hsa-miR-122-5p | MKLN1 |
| hsa-miR-122-5p | CHDH |
| hsa-miR-122-5p | FIG4 |
| hsa-miR-122-5p | SF3A1 |
| hsa-miR-122-5p | HS3ST1 |
| hsa-miR-122-5p | TSHZ2 |
| hsa-miR-122-5p | THAP2 |
| hsa-miR-122-5p | TAS2R5 |
| hsa-miR-122-5p | KAT7 |
| hsa-miR-122-5p | CYB5D1 |
| hsa-miR-122-5p | F2RL1 |
| hsa-miR-122-5p | CINP |
| hsa-miR-122-5p | UNC13A |
| hsa-miR-122-5p | ADM2 |
| hsa-miR-122-5p | RFC2 |
| hsa-miR-122-5p | WDR31 |
| hsa-miR-122-5p | NOM1 |
| hsa-miR-122-5p | NARS |
| hsa-miR-122-5p | TERF2 |
| hsa-miR-122-5p | NIP7 |
| hsa-miR-122-5p | ARHGEF5 |
| hsa-miR-122-5p | ZBTB8A |
| hsa-miR-122-5p | SMTNL2 |
| hsa-miR-122-5p | PSMB5 |
| hsa-miR-122-5p | NKAP |
| hsa-miR-122-5p | CYP20A1 |
| hsa-miR-122-5p | CENPM |
| hsa-miR-122-5p | IL2RA |
| hsa-miR-122-5p | RPS15A |
| hsa-miR-122-5p | PDP2 |
| hsa-miR-122-5p | DUSP18 |
| hsa-miR-122-5p | CXorf21 |
| hsa-miR-122-5p | ZNF573 |
| hsa-miR-122-5p | SLC1A5 |
| hsa-miR-122-5p | PGBD5 |
| hsa-miR-122-5p | SGOL1 |
| hsa-miR-122-5p | KIAA1919 |
| hsa-miR-122-5p | MYH11 |
| hsa-miR-122-5p | QPRT |
| hsa-miR-122-5p | BMS1 |
| hsa-miR-122-5p | RNF157 |
| hsa-miR-122-5p | LRCH3 |
| hsa-miR-122-5p | FMN1 |
| hsa-miR-122-5p | SLC16A5 |
| hsa-miR-122-5p | BAMBI |
| hsa-miR-122-5p | ROMO1 |
| hsa-miR-122-5p | IBA57 |
| hsa-miR-122-5p | RGS9BP |
| hsa-miR-122-5p | CEP89 |
| hsa-miR-122-5p | OLA1 |
| hsa-miR-122-5p | LILRA2 |
| hsa-miR-122-5p | PLXDC2 |
| hsa-miR-122-5p | ENTPD1 |
| hsa-miR-122-5p | PTGR2 |
| hsa-miR-122-5p | PTPLAD2 |
| hsa-miR-122-5p | FAM71F2 |
| hsa-miR-122-5p | SLC35E3 |
| hsa-miR-122-5p | TMCO1 |
| hsa-miR-122-5p | SLC35E2B |
| hsa-miR-122-5p | ACP6 |
| hsa-miR-122-5p | FAM217B |
| hsa-miR-122-5p | ADCY2 |
| hsa-miR-122-5p | SEC23B |
| hsa-miR-122-5p | NFX1 |
| hsa-miR-122-5p | FADS6 |
| hsa-miR-122-5p | PIGG |
| hsa-miR-122-5p | ABCA6 |
| hsa-miR-122-5p | DNPEP |
| hsa-miR-122-5p | TEP1 |
| hsa-miR-122-5p | XIAP |
| hsa-miR-122-5p | TMEM40 |
| hsa-miR-122-5p | SLC25A33 |
| hsa-miR-122-5p | RPS6KA5 |
| hsa-miR-122-5p | PXMP4 |
| hsa-miR-122-5p | MAP4K2 |
| hsa-miR-122-5p | DPY19L4 |
| hsa-miR-122-5p | ADRBK2 |
| hsa-miR-122-5p | SPIB |
| hsa-miR-122-5p | ARL17B |
| hsa-miR-122-5p | LIN52 |
| hsa-miR-122-5p | PGBD4 |
| hsa-miR-122-5p | MTAP |
| hsa-miR-122-5p | LRRC3C |
| hsa-miR-122-5p | OMD |
| hsa-miR-122-5p | MED18 |
| hsa-miR-122-5p | JPH2 |
| hsa-miR-122-5p | ZNF74 |
| hsa-miR-122-5p | IYD |
| hsa-miR-122-5p | MASTL |
| hsa-miR-122-5p | CCR6 |
| hsa-miR-122-5p | MRI1 |
| hsa-miR-122-5p | ZNF786 |
| hsa-miR-122-5p | C16orf45 |
| hsa-miR-122-5p | TIGD6 |
| hsa-miR-122-5p | ZYG11B |
| hsa-miR-122-5p | PTCD3 |
| hsa-miR-122-5p | TDRD1 |
| hsa-miR-122-5p | ESF1 |
| hsa-miR-122-5p | XKR4 |
| hsa-miR-122-5p | WDR17 |
| hsa-miR-122-5p | VPS53 |
| hsa-miR-122-5p | SSTR2 |
| hsa-miR-122-5p | SLC31A1 |
| hsa-miR-122-5p | SLC16A10 |
| hsa-miR-122-5p | SBNO1 |
| hsa-miR-122-5p | RBM23 |
| hsa-miR-122-5p | RBL1 |
| hsa-miR-122-5p | RAB10 |
| hsa-miR-122-5p | PNRC1 |
| hsa-miR-122-5p | NFE2L1 |
| hsa-miR-122-5p | MPLKIP |
| hsa-miR-122-5p | MAPK1 |
| hsa-miR-122-5p | LRAT |
| hsa-miR-122-5p | KDELR1 |
| hsa-miR-122-5p | GMEB1 |
| hsa-miR-122-5p | GK5 |
| hsa-miR-122-5p | GDE1 |
| hsa-miR-122-5p | STXBP2 |
| hsa-miR-122-5p | ESYT2 |
| hsa-miR-122-5p | C17orf85 |
| hsa-miR-122-5p | ALG14 |
| hsa-miR-122-5p | CNDP1 |
| hsa-miR-122-5p | BROX |
| hsa-miR-122-5p | TECPR1 |
| hsa-miR-122-5p | CCDC142 |
| hsa-miR-122-5p | ZNF841 |
| hsa-miR-122-5p | SMG1 |
| hsa-miR-122-5p | FLYWCH2 |
| hsa-miR-122-5p | SLC38A9 |
| hsa-miR-122-5p | LIMS1 |
| hsa-miR-122-5p | PLEKHS1 |
| hsa-miR-122-5p | F2 |
| hsa-miR-122-5p | DCAF7 |
| hsa-miR-122-5p | SIK2 |
| hsa-miR-122-5p | GP2 |
| hsa-miR-122-5p | POLR2D |
| hsa-miR-122-5p | NKPD1 |
| hsa-miR-122-5p | KRBA2 |
| hsa-miR-122-5p | ZNF655 |
| hsa-miR-122-5p | SYNPO2L |
| hsa-miR-122-5p | TMEM56 |
| hsa-miR-122-5p | INO80 |
| hsa-miR-122-5p | RNF19B |
| hsa-miR-122-5p | KDELC2 |
| hsa-miR-122-5p | KIF1C |
| hsa-miR-122-5p | TCF23 |
| hsa-miR-122-5p | MRPL17 |
| hsa-miR-122-5p | FAM120AOS |
| hsa-miR-122-5p | NAGK |
| hsa-miR-122-5p | KCMF1 |
| hsa-miR-122-5p | PRR23A |
| hsa-miR-122-5p | SNTB2 |
| hsa-miR-122-5p | MOGAT1 |
| hsa-miR-122-5p | CLK4 |
| hsa-miR-122-5p | ZNF431 |
| hsa-miR-122-5p | WWC1 |
| hsa-miR-122-5p | C9orf69 |
| hsa-miR-122-5p | PLEKHM3 |
| hsa-miR-122-5p | VSIG1 |
| hsa-miR-122-5p | ARIH2OS |
| hsa-miR-122-5p | TMEM168 |
| hsa-miR-122-5p | ALG1 |
| hsa-miR-122-5p | ZNF281 |
| hsa-miR-122-5p | C3 |
| hsa-miR-122-5p | FLG2 |
| hsa-miR-122-5p | SAR1A |
| hsa-miR-122-5p | TMEM180 |
| hsa-miR-122-5p | KIAA1143 |
| hsa-miR-122-5p | KCNA7 |
| hsa-miR-122-5p | NASP |
| hsa-miR-122-5p | BCL2L15 |
| hsa-miR-122-5p | C3orf62 |
| hsa-miR-122-5p | SLC35E1 |
| hsa-miR-122-5p | RUNDC1 |
| hsa-miR-122-5p | PTPDC1 |
| hsa-miR-122-5p | MRPS25 |
| hsa-miR-122-5p | LYRM4 |
| hsa-miR-122-5p | ACOT9 |
| hsa-miR-122-5p | RNF216 |
| hsa-miR-122-5p | COL13A1 |
| hsa-miR-122-5p | GPR155 |
| hsa-miR-122-5p | ANKRD36 |
| hsa-miR-122-5p | DNAJB13 |
| hsa-miR-122-5p | MAFF |
| hsa-miR-122-5p | ST3GAL1 |
| hsa-miR-122-5p | THAP6 |
| hsa-miR-122-5p | FANCC |
| hsa-miR-122-5p | XPO5 |
| hsa-miR-122-5p | RRP36 |
| hsa-miR-122-5p | ACTR5 |
| hsa-miR-122-5p | MED29 |
| hsa-miR-122-5p | SPECC1 |
| hsa-miR-122-5p | SPTLC3 |
| hsa-miR-122-5p | IFNAR2 |
| hsa-miR-122-5p | MED7 |
| hsa-miR-122-5p | KIF6 |
| hsa-miR-122-5p | P3H4 |
| hsa-miR-122-5p | ARPIN |
| hsa-miR-122-5p | HACD4 |
| hsa-miR-122-5p | MAP1B |
| hsa-miR-122-5p | FBXO6 |
| hsa-miR-122-5p | MAPK8IP2 |
