# Supplementary figures and images for: The Inhibition of Fibrosis and Inflammation in Obstructive Kidney Injury via the miR-122-5p/SOX2 Axis Using USC-Exos
Source: Biomater Res. 2024 Apr 10;28:0013. doi: 10.34133/bmr.0013 (PMC11014086; doi:10.34133/bmr.0013)

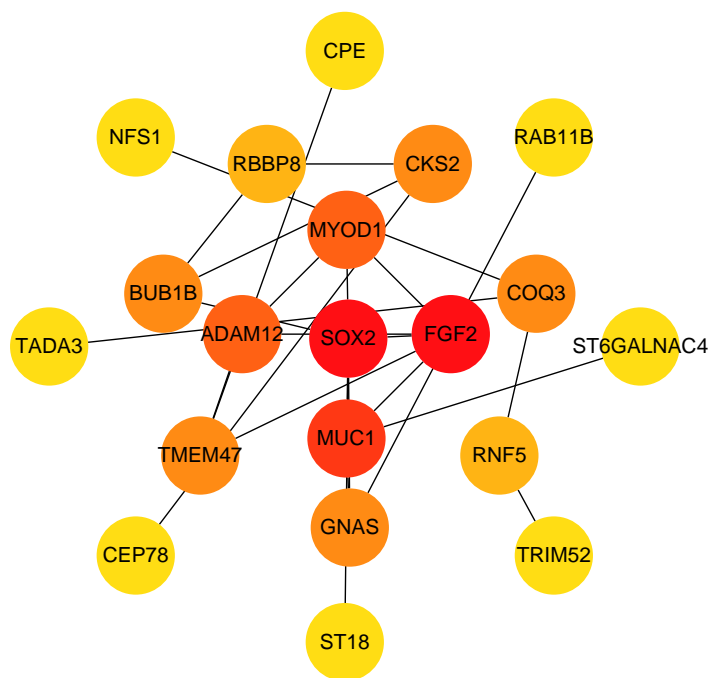

Supplement: Supplementary 1 — Fig. S1 Tables S1 to S4 [file bmr.0013.f1.zip › Supplementary Fig 1.pdf]
